# Abnormal Mammary Development in 129:STAT1-Null Mice is Stroma-Dependent

**DOI:** 10.1371/journal.pone.0129895

**Published:** 2015-06-15

**Authors:** Jane Q. Chen, Hidetoshi Mori, Robert D. Cardiff, Josephine F. Trott, Russell C. Hovey, Neil E. Hubbard, Jesse A. Engelberg, Clifford G. Tepper, Brandon J. Willis, Imran H. Khan, Resmi K. Ravindran, Szeman R. Chan, Robert D. Schreiber, Alexander D. Borowsky

**Affiliations:** 1 Center for Comparative Medicine, University of California, Davis, California, United States of America; 2 Department of Animal Science, University of California, Davis, California, United States of America; 3 Division of Basic Sciences, Cancer Center and Department of Biochemistry and Molecular Medicine, University of California, Davis, School of Medicine, Sacramento, California, United States of America; 4 Mouse Biology Program, University of California, Davis, California, United States of America; 5 Department of Pathology and Immunology, Washington University School of Medicine, St. Louis, Missouri, United States of America; 6 Department of Pathology and Laboratory Medicine, University of California, Davis, School of Medicine, Sacramento, California, United States of America; University of Tennessee Health Science Center, UNITED STATES

## Abstract

Female 129:*Stat1-null* mice (129S6/SvEvTac-*Stat1^tm1Rds^* homozygous) uniquely develop estrogen-receptor (ER)-positive mammary tumors. Herein we report that the mammary glands (MG) of these mice have altered growth and development with abnormal terminal end buds alongside defective branching morphogenesis and ductal elongation. We also find that the 129:*Stat1-null* mammary fat pad (MFP) fails to sustain the growth of 129S6/SvEv wild-type and *Stat1-null* epithelium. These abnormalities are partially reversed by elevated serum progesterone and prolactin whereas transplantation of wild-type bone marrow into 129:*Stat1-null* mice does not reverse the MG developmental defects. Medium conditioned by 129:*Stat1-null* epithelium-cleared MFP does not stimulate epithelial proliferation, whereas it is stimulated by medium conditioned by epithelium-cleared MFP from either wild-type or 129:*Stat1-null* females having elevated progesterone and prolactin. Microarrays and multiplexed cytokine assays reveal that the MG of 129:*Stat1-null* mice has lower levels of growth factors that have been implicated in normal MG growth and development. Transplanted 129:*Stat1-null* tumors and their isolated cells also grow slower in 129:*Stat1-null* MG compared to wild-type recipient MG. These studies demonstrate that growth of normal and neoplastic 129:*Stat1-null* epithelium is dependent on the hormonal milieu and on factors from the mammary stroma such as cytokines. While the individual or combined effects of these factors remains to be resolved, our data supports the role of STAT1 in maintaining a tumor-suppressive MG microenvironment.

## Introduction

The microenvironment of the mammary gland (MG) stromal is a complex mixture of cells, tissues and molecules that is essential for normal growth and development of the glandular epithelium [1–3]. Development of the MG is also stimulated by hormones acting on the epithelium and the surrounding stroma [4, 5]. The Janus Kinase (JAK)-Signal Transducer and Activator of Transcription (STAT) pathway plays a central role in this development as a primary intermediate for growth factor-, cytokine- and hormone-induced signaling [6, 7]. Among the various STAT molecules, STAT5a and STAT5b play a central role in MG development and lactation [8, 9]. By contrast, epithelial STAT3 plays a crucial role during involution [10] while STAT6 has been implicated during gestation-induced MG growth [11]. On the other hand, STAT1 appears to be functional only in the MG of nulliparous and post-lactational female mice [6].

The functional analysis of STAT3, STAT5 and STAT6 in the MG has benefited from a range of genetically modified mouse models [6]. While several models of STAT1 function have been developed, these have focused primarily on interferon-γ (IFNγ) signal transduction and transcription. Studies of MG development in mice in the absence of STAT1, either through germline knockout [12] or somatic knockout conditional to the mammary epithelium [13], did not identify abnormalities in ductal branching or elongation. However, those studies did not review the ontogeny of MG development, lactation and involution. Further, developmental endpoints were not the primary objective of those analyses that focused on tumorigenesis [6].

Studies of mammary tumorigenesis in female *Stat1* knockout (*Stat1-null*) mice describe a potential tumor suppressor role for STAT1. Indeed, four separate groups have shown that *Stat1-null* mice have an increased susceptibility to mammary tumors in a variety of contexts [12–15]. Two laboratories used C.129S6(Cg)-*Stat1* ^tm1Dlv^ mice bearing the *Stat1*-null allele backcrossed to the Balb/c strain to demonstrate that STAT1 functions as a mammary tumor suppressor [15, 16]. In those studies tumor formation was induced either via an additional oncogene or repeated breeding [15, 16]. Klover et al. developed a targeted mammary epithelial *Stat1* knockout which they crossed with FVB:cNeu to demonstrate epithelial cell intrinsic tumor suppression by STAT1 [13].

The 129S6/SvEvTac-*Stat1* ^*tm1Rds*^ homozygous female mice used herein (129:*Stat1-null* hereafter) [17] spontaneously develop distinctive estrogen receptor (ER) positive mammary tumors that mimic human luminal intrinsic subtypes of breast cancer [12]. These spontaneous tumors arise late (latency 18–20 months of age), are ~50% penetrant and have a consistent and unique phenotype different from the histopathological patterns seen in other tumor suppressor knockout models [18]. A potential mechanism for the observed increased risk of MG carcinoma might stem from changes in the underlying MG during development and aging. We hypothesized that the MG of these mice undergo altered development that reflects an altered stromal microenvironment. To this end, we initiated a rigorous study of MG development and the contribution of the stromal microenvironment in 129:*Stat1-null* females. Experimental dissection and recombination of various elements of the MG and mammary fat pad (MFP) demonstrated a strong effect of the host environment and a less pronounced epithelial defect in branching morphogenesis. The loss of STAT1 affected both systemic and local host factors that contributed to the epithelial abnormalities.

Given that many mouse models of mammary tumorigenesis exhibit abnormal MG development at an early age [19], the results of this study provide critical clues to the roles of candidate genes during neoplastic progression [19, 20].

## Materials and Methods

### Mouse Model

129S6/SvEvTac-*Stat1* ^*tm1Rds*^ (129:*Stat1-null*) mice [17, 21, 22] were contributed by the Schreiber lab (RDS), and wild-type (WT) 129S6/SvEv (129SvEv WT) mice were purchased from Taconic Farms (Hudson, NY). All surgery was performed under Nembutal anesthesia (60 mg/kg) followed by post-surgical analgesia (Buprinex; 0.05 mg/kg). All mice were euthanized using an overdose of Nembutal (120 mg/kg) prior to collection and fixation of tissues.

### Histopathology and Whole Mount Preparation

Tissues were fixed in 10% neutral buffered formalin at room temperature for 24 h then placed in 70% ethanol until processing, which was normally within 24 h. A Tissue-Tek VIP autoprocessor (Sakura, Torrance, CA) was used to process tissues that were then embedded in Paraplast (melting temperature 56–60°C), sectioned to 4 μm and mounted on glass slides. Sections were stained with hematoxylin and eosin (H&E) for pathologic analysis. For MG whole mount preparation, the MFPs were dissected, placed onto a glass slide and fixed in 10% neutral buffered formalin. Tissues were further processed in a tissue cassette starting with 70% alcohol for 2 h then transferred to 100% alcohol for another 2 h. Glands were then defatted using three changes of xylene (30 min, 1 h, 1 h) and then rehydrated through a graded series of alcohol. After rinsing in running tap water for 30 min, the tissues were stained with hematoxylin for 2 min. Glands were destained in a 1% HCl solution for 15 min, then were placed under running tap water for approximately 30 min, 70% alcohol for 1 h, 100% alcohol for 1 h, and finally xylene for 1 h. Whole mounts were then submerged in methyl salicylate for storage.

### Morphological Analyses

**Fig 1:**
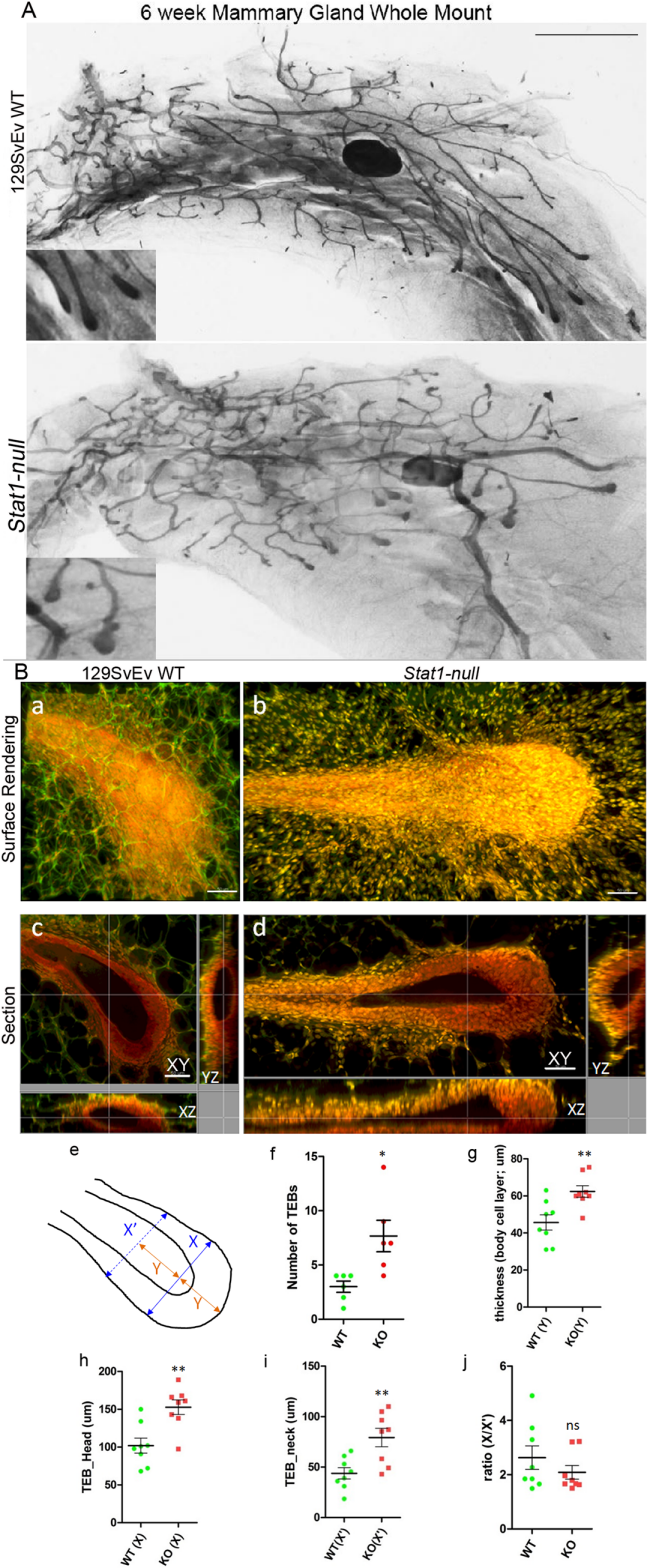
Six week 129:*Stat1-null* mammary glands have more and larger TEBs. Hematoxylin stained whole mount images of inguinal mammary glands from 6-week-old 129SvEv WT and 129:*Stat1-null* (*Stat1-null*) mice (A). Insets illustrate alterations in TEB between 129SvEv WT and *Stat1-null* mice. Scale bar is 2 mm. Three-dimensional visualization of TEBs from whole mount MG of 6-week-old 129SvEv WT and 129:*Stat1-null* mice using autofluorescence with laser scanning confocal microscopy (B). Surface rendered 3D images (Ba, Bb) and the orthogonal sections of XY, XZ and YZ planes (Bc, Bd) are shown. Panels on left (Ba, Bc) are from 129SvEv WT MGs and right (Bb, Bd) are from 129:*Stat1-null*. Red signal (nuclei; carmine) and green signal (cell; autofluorescence) are indicated in images. Scale bar is 50 μm. The schematic (Be) indicates the measuring points for comparing parameters in 3D images of TEBs. The widest TEB diameter is X (TEB head length). The thickness of TEB at the tip equals Y (body cell layer thickness). X’ (TEB neck length) was measured at the point equal 2xY from the tip (Bf). TEBs were quantitated when the X > 100 μm. All data are mean ± SEM (n = 5). *P = 0.0128. 129:*Stat1-null* gland TEBs (KO) are larger in size as measured by body layer thickness (Bg, **P = 0.0058) head size (h, **P = 0.0025) and neck size (I, **P = 0.0050). n = 8. However, the differences in the ratio (X/X’) are not significant (Bj).

Structural differences in terminal end buds (TEBs) were visualized using a laser scanning based imaging method described previously [23]. In short, MGs from 129 WT and 129:*Stat1-null* were spread on glass slides, fixed with 4% paraformaldehyde in phosphate-buffered saline (PBS) for 15 min then further fixed overnight in Carnoy’s solution (75% ethanol, 25% acetic acid). Tissues were stained with carmine alum solution, overnight, followed by destaining with acidic EtOH (70% EtOH, 0.6 M HCl) for ~2 h until whole mounts showed good contrast. Whole mounts were dehydrated (30 min each for 80%, 90%, 95% and 100% EtOH) and defatted in xylene overnight prior to mounting with Permount (Electron Microscopy Sciences). To capture a three-dimensional image of TEBs, whole mounts were assessed using laser scanning-based tissue autofluorescence/fluorescence imaging (LS-TAFI) with an LSM 710 (Carl Zeiss Microscopy, Ltd) set to “main dichroic beam splitter” (MBS; 488/561 nm) and “detection range” (495 to 553 nm for MBS 488 nm; 568 to 728 nm for MBS 561 nm). Scanned images were analyzed with IMARIS (Bitplane, South Windsor, CT) to quantify each parameter indicated in Fig 1. Total duct length and branch points were measured on images of MG whole mounts stained with either hematoxylin or carmine. To measure total duct length, greyscale images were inverted and analyzed with IMARIS imaging software (Bitplane) using the filament tracing feature.

### Microarray Analysis

Microarray analysis (accession number GSE63025) met the 6 critical elements for Minimum Information About a Microarray Experiment (http://www.mged.org/Workgroups/MIAME/miame.html). Total cellular RNA was isolated from flash-frozen MGs using TRIzol reagent (Life Technologies) and a modified protocol that incorporates a second extraction with acid phenol/chloroform/isoamyl alcohol (25:24:1, pH 4.3). Total RNA quantity and quality were assessed on a NanoDrop spectrophotometer (Thermo Scientific, Waltham, MA) and an Agilent 2100 Bioanalyzer (Agilent Technologies, Santa Clara, CA), respectively. Microarray gene expression profiling (at the UC Davis Comprehensive Cancer Center’s Genomics Shared Resource) was performed by whole-transcript analysis on Affymetrix GeneChip Mouse Gene 1.0 Sense Target (ST) Arrays, which provide coverage for a total of 26,166 RefSeq transcripts from 21,041 individual genes. Briefly, biotinylated sense strand DNA targets were prepared from 100 ng total RNA using the Ambion WT Expression and Affymetrix GeneChip WT Terminal Labeling Kits according to the manufacturers’ protocols. All downstream microarray processing procedures, including hybridization, washing, staining, and array scanning were performed according to Affymetrix’s standard protocols. Microarray data analysis was performed with GeneSpring GX software (Agilent Technologies). Microarray probe intensity values (CEL files) were background-corrected, summarized, and normalized using the Robust Multi-array Average (RMA16) algorithm [24] and filtered on raw intensity values for probe sets that exceeded a threshold of >38. Comparison analysis was performed to identify genes that were differentially expressed (≥1.5-fold) between the inguinal MFP of 129:*Stat1-null* and 129SvEv WT mice. Biological interpretation of the resulting gene list was performed using the functional annotation and clustering tools available at the Database for Annotation, Visualization and Integrated Discovery (DAVID) Bioinformatics Resources 6.7 [25]. In brief, these determine gene-gene functional relationships with kappa statistics [26], a novel agglomeration algorithm to organize them into biological modules, and then to calculate enrichment scores based on Fisher’s Exact Test [25].

### Heat Mapping

Heat mapping was performed with Heat map Builder [27]. The 50 genes with a 2-fold difference in expression that were either ‘high in 129SvEv WT’ or ‘high in *Stat1-null*’ as measured by microarray were heat mapped and color-coded with the ranges indicated on the figure. The cytokine protein profile was also heat mapped to compare the levels of each cytokine.

### Mammary Epithelial Tissue Transplantation

In order to provide host transplantation sites, the inguinal MFPs of three-week old 129SvEv WT and 129:*Stat1-null* female mice were “cleared” of epithelial tissue as described [28]. Explants of epithelium-containing MG (1–2 mm pieces) from donor 129SvEv WT or 129:*Stat1-null* mice were dissected from the inguinal MG (between the nipple and lymph node) and transplanted into the epithelium-cleared mammary fat pads (ECFP) of host mice (left ECFP received 129:*Stat1*-null tissue and right ECFP received 129SvEv WT tissue).

### Hormone Treatment

Mice were administered daily SC injections of progesterone (P, 0.5 mg, Acros Organics, New Jersey) and/or domperidone (DOM, 1.0 mg/Kg, Sigma Chemical Co, St. Louis, MO; to induce hyperprolactinemia), or estrogen (E, 1 μg, 17β estradiol, Sigma) for 14 d. Both P and E were suspended in sterile sesame oil, while DOM was dissolved in sterile saline (pH 3.0). Control animals were injected with sesame oil and saline. All animals received the same amount of sesame oil and saline, every 24 h, for 14 d.

### Bone Marrow Grafting

Three week old recipient mice (129SvEv WT or 129:*Stat1-null*) were irradiated with 1000 rads in a Model 143–68 Blood Component Irradiator (J.L. Shepard, San Fernando, CA). Bone marrow was collected from dissected femurs and tibias of non-irradiated donor mice that had been given a lethal dose of Nembutal (120 mg/Kg). Bone marrow was flushed into 14 mL culture tubes containing 5 mL of Hank’s balanced salt solution (HBSS) on ice using an 18 gauge needle and 3 mL syringe. Crude bone marrow was centrifuged then resuspended in 1 mL of ACK (ammonium-chloride-potassium) lysing buffer (Gibco, Grand Island, NY) for ~1 min. Samples from the same donor genotype (129SvEv WT or 129:*Stat1-null*) were combined, resuspended in 3 mL HBSS and filtered through a 35 μm filter (BD Biosciences, San Jose, CA). Viable cells were counted using trypan blue stain. Bone marrow cells were resuspended in sterile PBS (2 x 10^6^ cells/100 μl) for injections into irradiated recipients. Recipients were injected via tail vein with 200 μL of bone marrow preparation from WT or 129:*Stat1-null* mice. After injection, recipient mice were given Sulfatrim PO with an active dose between 8–24 mg/kg/d for a period of 10 d. Bone marrow eradication and “take” were documented by qPCR of gDNA (see S1 Text for details of materials and methods) from peripheral blood, which showed that all surviving animals had complete engraftment of the donor marrow without residual host marrow (data not shown).

### Immunohistochemistry (IHC)

Antigen retrieval was performed for 45 min with citrate buffer at pH 6.0 in a Decloaking Chamber (Biocare Medical, Concord, CA) at 125°C and 15 psi. Slides were blocked with normal goat serum then incubated with a rabbit monoclonal anti-CD3 (1:1000; Clone [Sp7], Catalog number Ab16669, Batch number GR125527-1, Abcam, Cambridge, MA) overnight at room temperature in a humidified chamber, followed by a biotinylated goat anti-rabbit secondary antibody (1:1000). The Vectastain ABC Kit Elite Kit and a diaminobenzidine Peroxidase Substrate Kit (Vector Labs, Burlingame, CA) were used for amplification and visualization of signal, respectively. Mouse spleen and thymus were used as positive controls for CD3 staining.

### Tumor Biopsy and Cell Transplantation

For tumor growth studies, biopsies (1–2 mm pieces) of primary tumors from 129:*Stat1-null* mice were transplanted into the intact inguinal MG of 6–8 week old 129SvEv WT or 129:*Stat1-null* mice. Following transplantation, mice were palpated twice weekly to monitor for tumor appearance. For transplantation of a 129:*Stat1-null* mammary tumor cell line, SSM2 cells [12] (hereafter designated SSM2^UCD^) were used. SSM2^UCD^ cells were grown in the appropriate culture media supplemented with 10% fetal bovine serum [12]. Nearly confluent (80%) cultures were trypsinized, washed 3 times with PBS, and counted. Cells were stored in liquid nitrogen until use and were of relatively low passage number (12–27). A bolus of previously cultured cells (3 x 10^4^) was injected into the uncleared inguinal MG of 6–8 week old 129SvEv WT or 129:*Stat1-null* mice. Two weeks after injection, tumors were removed and processed for histology and IHC.

### Conditioned Medium Preparation

129SvEv WT and 129:*Stat1-null* inguinal MGs were cleared at 3 weeks of age as described above. At 10 weeks of age, conditioned medium (CM) was prepared from the ECFPs of these mice by dicing them into fragments (~8 mm^3^) and incubating them in serum-free Dulbecco's modified Eagle medium (DMEM) (7.5 mg tissue/mL) for 48 h at 37°C. A basal medium was prepared by incubating serum-free DMEM for 48 h at 37°C in the absence of ECFP. Both CM and basal medium were subsequently filtered (0.22 μm) and either used immediately in experiments or stored at -20°C. Some CM and basal medium was concentrated using centrifugal filters (Amicon ultra, Millipore, Billerica, MA) for mouse cytokine/chemokine assay experiments, or for storage at -80°C.

Comma-1D cells were maintained in growth media (DMEM/F12, 2% fetal bovine serum, 10 μg/mL bovine insulin, 5 ng/mL recombinant human epidermal growth factor, penicillin/streptomycin). Cells (3000/well) were plated into 96-well plates 24 h before the start of the growth assay (day 0). A standard curve was created by plating known quantities of cells (0–50,000/well) before quantifying final cell number on day 0 using a methylene blue assay [29]. Following a wash with PBS, media were changed to basal media or CM on day 0, with another medium change on day 2. Cells were then fixed in 10% formalin, stained for 35 min with 1% methylene blue, washed using 0.01 M borate buffer (pH 8.5) and the blue stain eluted from the cells with a 1:1 mix of 95% ethanol and 0.1 M HCl. The plates were read at 665 nm. Blank wells did not contain cells but were subjected to the entire fixing and staining process, and their average absorbance subtracted from all wells.

### Multiplex Cytokine/Chemokine Assay

A Milliplex MAP 32-analyte kit for the analysis of mouse cytokines/chemokines (EMD Millipore) was used for measuring 32 cytokines and chemokines simultaneously in each sample. The MAP system is based on unique populations of 100 different, individually identifiable microbead sets coupled to an antibody specific to a cytokine or chemokine that captures the relevant analyte in the sample prior to detection using a second, biotinylated antibody specific to each respective analyte [30]. Multiplex assays were performed according to the manufacturer’s instructions. Data for each of the analytes were collected as median fluorescent intensity (MFI) that was used to calculate analyte concentrations in pg/mL using the software package BioPlex Manager 5.0 (Bio-Rad) as previously described [31].

### Statistical Analysis

Pairwise comparisons between means or least square means were analyzed by Student’s t test. Parameter means for ductal length and branching points were statistically compared using GraphPad Prism software (GraphPad Software, Inc., La Jolla, CA). For COMMA 1D cell proliferation studies, statistical significances were tested by 2-way ANOVA.

### Ethics Statement

Mice were maintained and housed in a UC Davis animal facility and handled in strict accordance with the guidelines described by the Association for Assessment and Accreditation of Laboratory Animal Care International and an Institutional Animal Care and Use Committee of the University of California, Davis approved protocol (protocol numbers 17604 and 16642).

## Results

### 129:*Stat1-null* Mice Exhibit Abnormal Mammary Gland Growth and Development

129:*Stat1-null* females are capable of nursing pups that thrive and have normal appearing milk in their opaque stomachs. Whole mounts of MGs from gestating and lactating females were grossly normal, where MGs from post-partum females had no obvious structural or developmental abnormalities (S1 Fig). By contrast, nulliparous 129:*Stat1-null* mice had delayed and abnormal MG growth and development relative to 129SvEv WT mice. Whole mounts of the MG from 129:*Stat1-null* mice at 6 weeks of age had larger and more abundant TEBs than the 129SvEv WT (Fig 1A) and showed greater variation in ductal elongation, ranging from quite stunted (not shown) to normal elongation (Fig 1A). LS-TAFI [23] revealed that the neck region of the TEB from 129:*Stat1-null* mice was surrounded with an extended zone of disorganized small stromal cells that were absent in the proximity of TEB from 129SvEv WT mice (Fig 1B). Quantitative analysis of three dimensional reconstructions verified that the TEB in 129:*Stat1-null* mice were more numerous (Fig 1B and 1F) and larger than in 129SvEv WT mice and had longer neck regions (Fig 1B, 1G, 1H and 1I).

**Fig 2:**
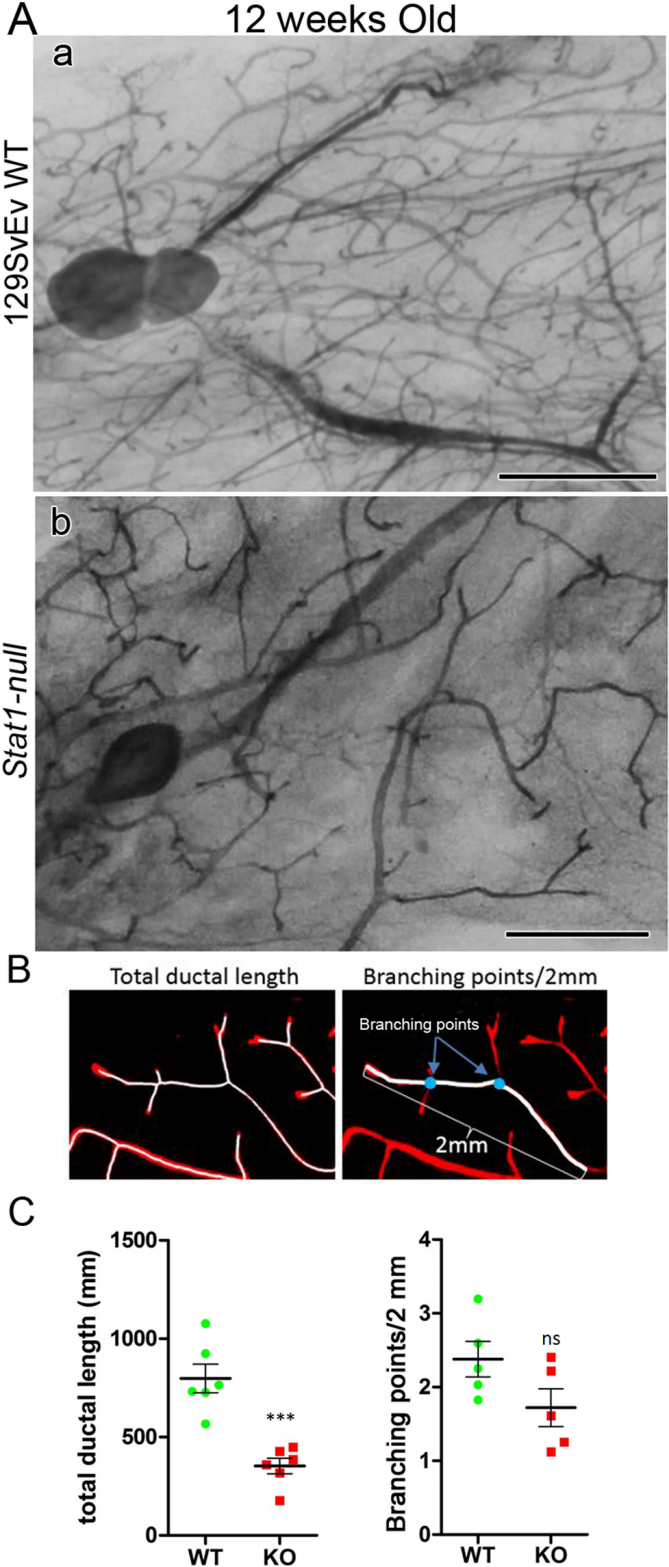
129:*Stat1-null* have diminished ductal length. Comparison between 129SvEv WT and 129:*Stat1-null* (*Stat1-null*) glands indicated that the *Stat1-null* glands had less ductal elongation. (A) Whole mount images of inguinal mammary gland from (a) 129SvEv WT and (b) *Stat1-null* (see S2 Fig for low power images of mammary gland whole mounts). Scale bar is 2 mm. (B) Total ductal length and branching points were measured on photo images of whole mount mammary glands stained with either hematoxylin or carmine red. For measuring total ductal length, images were analyzed with IMARIS imaging software using the filament tracing feature. Branching points were manually counted on ducts within 2 mm of end buds. (C) Quantitative analyses for total ductal length (left) and the number of branches (right) shows that *Stat1-null* (KO) glands have significantly (***P = 0.0003) shorter total ductal length. The number of branching points within 2 mm ductal ends was not significantly different between 129SvEv WT and *Stat1-null* glands. Data are mean ± SEM (n = 6).

In mammary gland whole mounts of mice at 12 weeks of age it was observed that all 129:*Stat1-null* MGs extended into their native MFPs but seemed to lag behind those from their 129SvEv WT counterparts, and rarely reached the end of the fat pad (S2 Fig). The 129SvEv WT MG appeared to have a higher density of ductal outgrowths and more branching than the 129:*Stat1-null* MG (Fig 2A) and so we tested this hypothesis by quantitative image analysis. This revealed a shorter overall length of the ductal network in the 129:*Stat1-null* MG (P = 0.0003) but no significant differences in the frequency of branch points in the developing 129SvEv WT and 129:*Stat1-null* MGs (Figs 2B and 1C).

Serum samples from selected cohorts were analyzed by the Vanderbilt University Mouse Hormone Core Laboratory for E, P, prolactin (PRL) and insulin levels. The differences between 129SvEv WT and 129:*Stat1-null* were not statistically significant (S3 Fig). Necropsies did not reveal significant gross or microscopic differences between the developing 129SvEv WT and 129:*Stat1-null* female pituitaries, ovaries and uteri (data not shown). The estrus cycle of each mouse was evaluated by examining the vaginal histology but had no apparent influence on mammary gland development.

### 129:*Stat1-Null* Mammary Fat Pad fails to support Epithelial Growth without Exogenous Hormones

**Fig 3:**
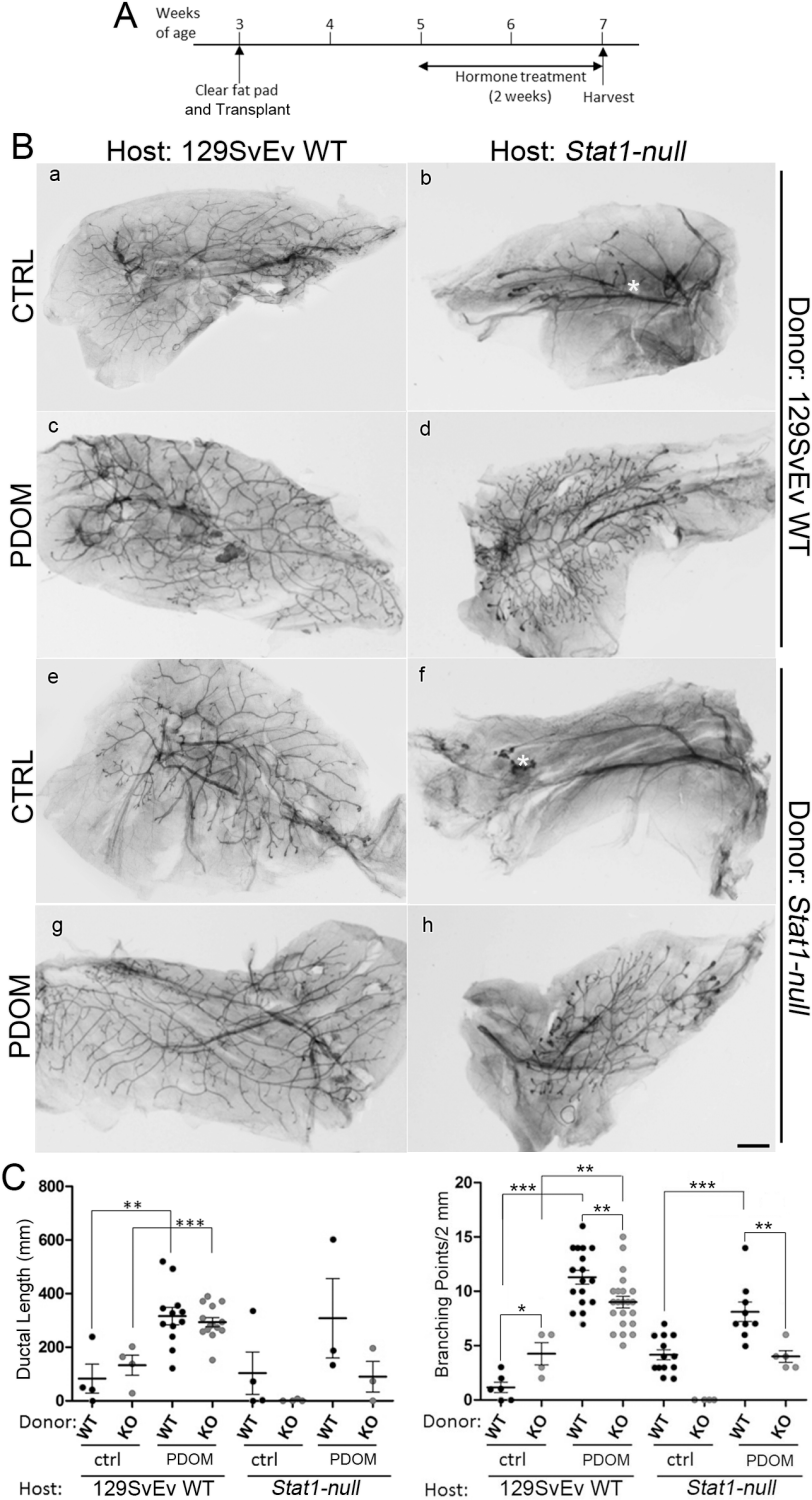
129:*Stat1-null* mammary fat pads impede epithelial transplant growth that is restored by exogenous hormones. Schematic depicting transplantation protocols between 129SvEv WT and *Stat1-null* glands and treatment with hormones (A). Inguinal mammary glands from 3-week-old host females were cleared of endogenous epithelium, while epithelium from donor 129SvEv WT and 129:*Stat1-null* (*Stat1-null*) mammary glands were transplanted into each epithelium-cleared fat pad in host mice 1 week after clearing surgery. Host mice were injected daily with PDOM from 5 to 7 weeks of age. Hematoxylin-stained whole mount images of reciprocally transplanted mammary glands with or without hormone treatment (B). Mammary glands in host 129SvEv WT (a, c, e, g) and *Stat1-null* (b, d, f, h) mice with donor epithelium from 129SvEv WT (a, b, c, d) and *Stat1-null* (e, f, g, h) mice are shown. Mammary glands from PDOM treated mice (c, d, g, h). White asterisks in b and f indicate approximate locations of transplantation sites. Quantitative analyses of the ductal network (C). The left panel graph indicates the total ductal length of mammary glands in each experiment (WT vs. KO for each treatment). Data are mean ± SEM, **P = 0.0026, ***P = 0.0005. The right panel graph is the analysis for the number of branching points. **P<0.01, ***P<0.001.

Heterologous transplantation was used to define the growth and developmental impacts of epithelial cell intrinsic factors versus the host microenvironment, including the MFP, bone marrow and endocrine/paracrine compartments. Simultaneous control autologous and cross “reciprocal” transplantation of 129SvEv WT and 129:*Stat1-null* mammary epithelium into 129SvEv WT (Fig 3A for experimental design) and 129:*Stat1-null* host ECFPs were performed with each recipient mouse hosting both 129SvEv WT and 129:*Stat1-null* epithelium.

Epithelial transplants were examined for ductal outgrowth 4 weeks after transplantation using hematoxylin stained whole mounts (Fig 3B). “Take” rates (successful transplants) and statistical comparisons between groups are recorded in S1 and S2 Tables, respectively. Transplants of 129SvEv WT donor and 129:*Stat1-null* donor epithelia grew into the mammary MFP when transplanted into the 129SvEv WT host (Panels a and e in Fig 3B). However, the 129:*Stat1-null* host supported fewer takes (S1 Table) and less outgrowth of the transplanted 129:*Stat1-null* donor epithelium as compared to the transplanted 129SvEv WT donor epithelium (Compare panels b and f in Fig 3B). Interestingly, the 129:*Stat1-null* epithelial transplants exhibited equal total duct length and significantly greater branching (P = 0.0157) in equal-sized fat pads in the 129SvEv WT ECFP compared to the autochthonous WT transplants (Fig 3C). This finding initially suggested that the 129:*Stat1-null* epithelium does not necessarily have an intrinsic defect. However, later studies using different experimental variables support an intrinsic epithelial defect.

Given that growth and development of the native MG in 129:*Stat1-null* females appeared to be rescued during pregnancy, and there is notable cooperatively between the actions of P and PRL during MG growth [32], the ability of P plus DOM (PDOM) to rescue the impaired growth of the transplants of 129:*Stat1-null* epithelium was examined. Exogenous E, P, DOM or PDOM were administered daily for two weeks to 129SvEv WT and 129:*Stat1-null* host mice bearing contralateral transplants of 129SvEv WT and 129:*Stat1-null* mammary epithelium.

Exogenous PDOM stimulated the growth of the donor MG epithelium in both 129SvEv WT and 129:*Stat1-null* hosts (Panels c, d, g, h in Fig 3B). The effect of E was similar to that induced by PDOM, whereas there was no effect when the host mice were treated with P or DOM alone (S4 Fig). Growth parameters (total ductal length and branching points) were enhanced in most transplants treated with PDOM (Fig 3C). Branching remained lower in 129:*Stat1-null* epithelium transplanted into either 129:*Stat1-null* or 129SvEv WT MFP even after PDOM treatment stimulated growth. This result suggests that the 129:*Stat1-null* mammary epithelium does have an intrinsic, albeit minor but statistically significant, defect in branching morphogenesis (Fig 3C). The results with PDOM treatment confirm, extend and reinforce the prior observations that pregnancy rescues the mammary phenotype in the native gland (S1 Fig).

### 129:*Stat1-null* MG Microarray Profile Differs from Wild-Type

**Fig 4:**
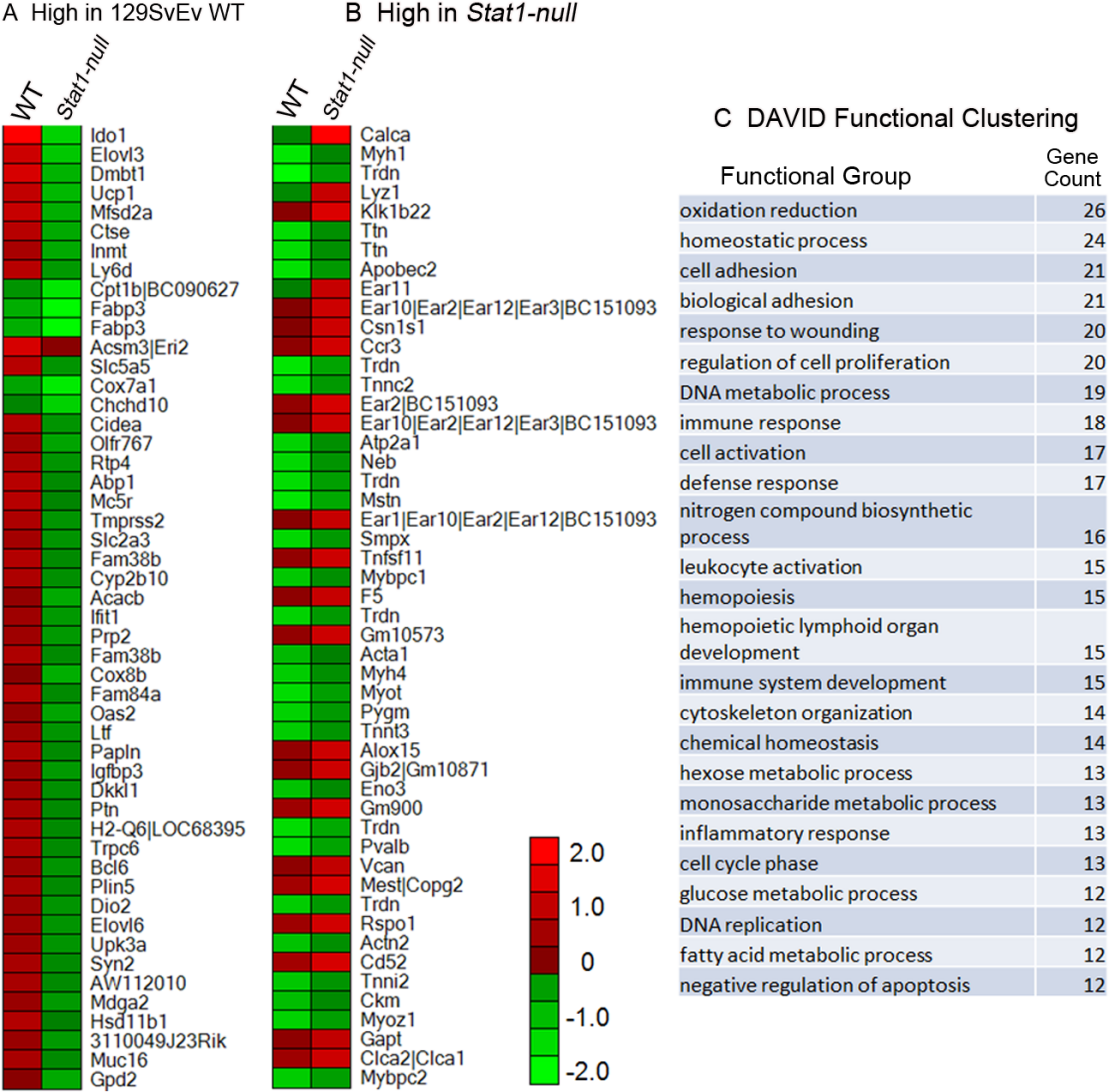
129:*Stat1-null* mammary gland expression microarray profiles indicate down-regulation of STAT1-related pathways with up-regulation of eosinophil genes. Gene expression microarrays were performed using RNA extracted from 129SvEv WT and 129:*Stat1-null* (*Stat1-null*) mammary glands (MG). The 50 genes > 2-fold higher in (A) 129SvEv WT MG and (B) *Stat1-null* MG are indicated in the heat maps (WT vs. *Stat1-null*). DAVID functional clustering (C) showed increased representation of immune cell related processes (immune response, leukocyte activation, immune system and development, inflammatory response, etc.), consistent with processes known to be affected by the loss of STAT1.

Microarray analysis revealed significant (≥1.5-fold) differences in mRNA expression for various genes in the MGs from either 129SvEv WT (Fig 4A) or 129:*Stat1-null* mice (Fig 4B), as can also be represented by heat maps. As expected, genes related to STAT1 function (e.g. IDO1, Indo1) had significantly lower expression in MGs from 129:*Stat1-null* mice. The expression of eosinophil associated ribonuclease (*Ear*) genes (*Ear1*, *Ear2*, *Ear10*, *Ear11*) was significantly higher in 129:*Stat1-null* MGs while the expression of genes relating to monocytes [33] also significantly differed between 129SvEv WT and 129:*Stat1-null*. The expression of calcitonin-associated peptide (*Calca*) mRNA had the highest elevation in 129:*Stat1-null* glands along with other genes related to reduction-oxidation pathways, suggesting metabolic deficiency or dysregulation. To further investigate which biological events are involved in the 129:*Stat1-null* glands, DAVID functional cluster analysis was performed (Fig 4C). The lists of genes from this analysis also highlighted the involvement of immune cell related activities in 129:*Stat1-null* mice (immune response, leukocyte activation, immune system development, inflammatory response, etc.) consistent with previous studies demonstrating that 129:*Stat1-null* mice are immunodeficient [34–36]. As expected, *Stat1* mRNA was markedly down-regulated while expression of various other *Stat* and *Jak* genes was differentially altered up and down when the 129:*Stat1-null* MG was compared with the 129SvEv WT (S5 Fig).

### Transplanted Wild-Type Bone Marrow does not Rescue 129:*Stat1-null* Epithelial Growth

**Fig 5:**
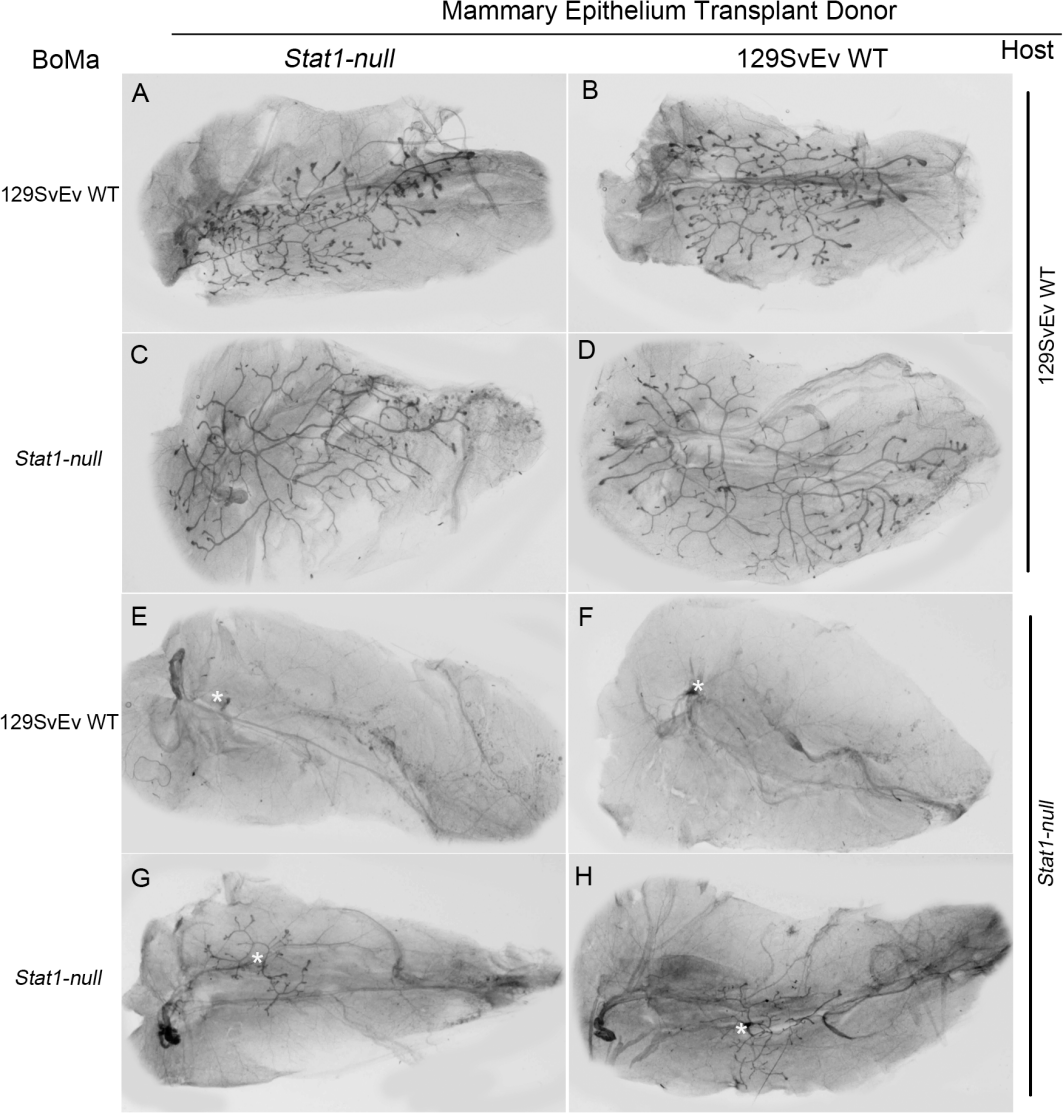
Bone marrow grafting does not alter 129:*Stat1-null* epithelial outgrowth. Hematoxylin-stained inguinal mammary gland whole mounts from 129SvEv WT and 129:*Stat1-null* (*Stat1-null*) mice. Briefly, lethally irradiated 129SvEv WT (A, B, C, D) and *Stat1-null* (E, F, G, H) were reconstituted with bone marrow (BoMa) from 129SvEv WT (A, B) or *Stat1-null* (C, D) and were transplant recipients for 129SvEv WT (B, D, F, H) and *Stat1-null* (A, C, E, G) mammary gland epithelium. The *Stat1-null* hosts show limited mammary transplant outgrowth, which did not improve with 129SvEv WT bone marrow transplantation. Additionally, no change in mammary gland outgrowth was seen in the 129SvEv WT hosts transplanted with *Stat1-null* bone marrow.

The 129:*Stat1-null* mice are immunologically impaired with known defects in natural killer (NK) cells and macrophages [34–36]. Cells derived from the bone marrow, including immune cells and particularly monocytes/macrophages, are known to facilitate MG development [37]. In order to determine if the 129:*Stat1-null* host defect could be attributed to bone marrow-derived cells, simultaneous bone marrow and MG reciprocal transplantations were performed (Fig 5). Briefly, lethally irradiated 129:*Stat1-null* mice were reconstituted with bone marrow from 129:*Stat1-null* (control) or 129SvEv WT mice, and then served as transplant recipients for contralateral 129:*Stat1-null* and 129SvEv WT epithelial MG transplants. Similarly, irradiated 129SvEv WT mice were reconstituted with either 129SvEv WT (control) or 129:*Stat1-null* bone marrow, and then served as recipients for simultaneous 129:*Stat1-null* and 129SvEv WT epithelial MG transplants.

All surviving animals had complete engraftment of the donor marrow without residual host marrow (data not shown). The 129:*Stat1-null* hosts showed limited growth of the transplanted mammary epithelium, which did not improve with transplantation of 129SvEv WT bone marrow. Additionally, no change in MG growth was recorded in the 129SvEv WT hosts transplanted with 129:*Stat1-null* bone marrow (Fig 5). Taken together, these experiments indicated that the mammary phenotype could not be rescued or created using transplanted bone marrow alone.

### Growth of Neoplastic Transplants is Impaired in 129:*Stat1-null* Hosts

**Fig 6:**
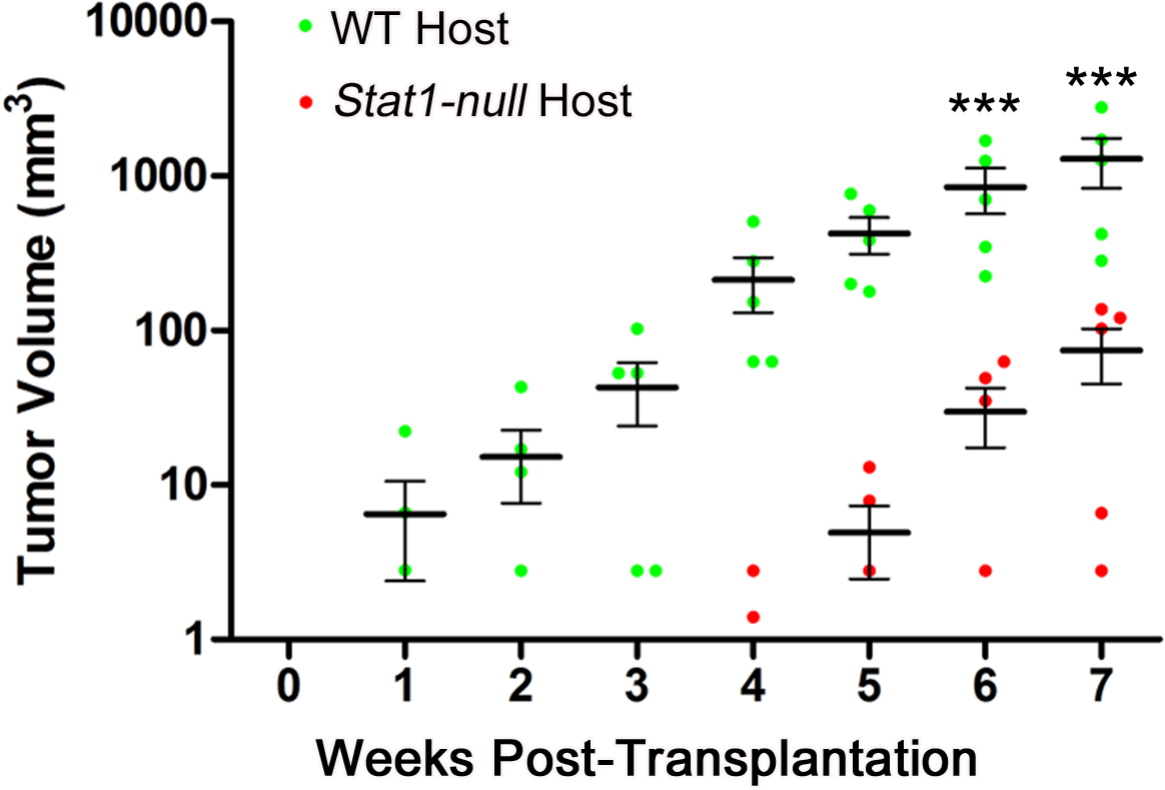
129:S*tat1-null* fat pads poorly support growth of transplanted tumors. Tumor volume changes in mammary glands of 129SvEv WT or 129:*Stat1-null* (*Stat1-null*) host mice over time. Explants of three primary mammary gland tumors from *Stat1-null* mice were transplanted into inguinal mammary glands of five 129SvEv WT or *Stat1-null* hosts. Tumors were measured by caliper twice weekly for 7 weeks. Tumor volumes in 129SvEv WT mice increased 10-fold faster than those in *Stat1-null* hosts. Data are mean ± SEM (n = 5), ***P<0.001. Each horizontal black bar represents the mean of tumor volumes.

STAT1 was previously shown to function as a tumor suppressor [12–16, 38]. Given our data indicating that STAT1 functions in the MG microenvironment to regulate growth of the normal epithelium, we performed experiments to assess the effects of this microenvironment on tumor cell growth. Three different primary MG tumors that arose in 129:*Stat1-null* mice were transplanted bilaterally into inguinal MFPs of 129SvEv WT and 129:*Stat1-null* recipient mice. Seven weeks post transplantation, the volume of resulting MG tumors in 129SvEv WT hosts was almost 10 times that of tumors grown in 129:*Stat1-null* hosts (Fig 6). Thus, the 129:*Stat1-null* local or systemic environment poorly supports the growth of transplanted primary tumor cells and suggests that the tumor suppressor action of STAT1 in the microenvironment/host is not functional or effective.

**Fig 7:**
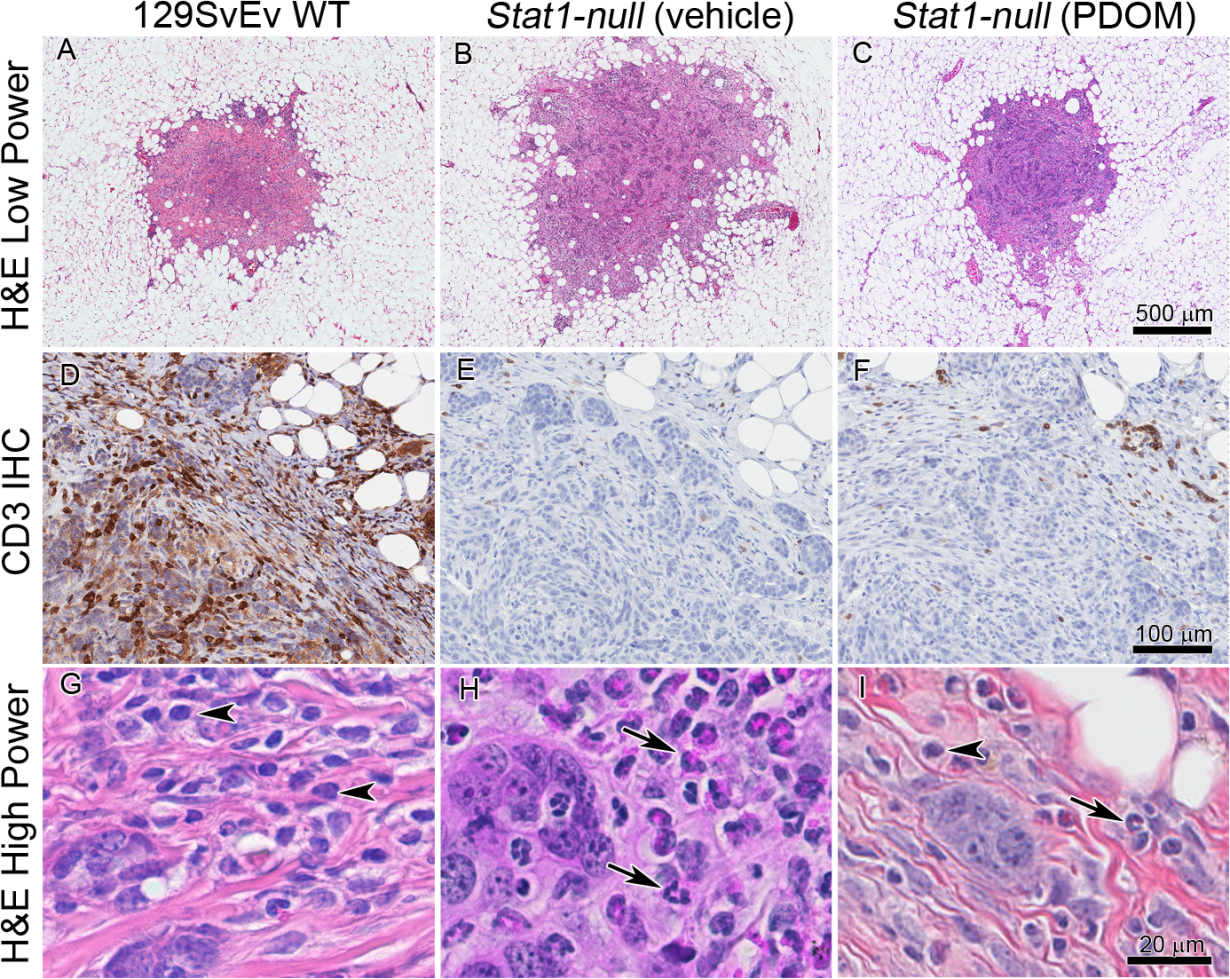
Differing host responses between 129:*Stat1-null* and 129SvEv WT to transplanted tumor cells. The SSM2^UCD^ tumor cell line derived from a 129:*Stat1-null* (*Stat1-null*) mammary gland tumor was transplanted into intact mammary glands of 6-week-old 129SvEv WT (A, D, G), *Stat1-null* mammary glands (B, E, H), and mammary glands of *Stat1-null* mice treated with PDOM (C, F, I). The SSM2 tumors were harvested and processed for histopathological review two weeks after transplantation. Upper 3 panels (A, B, C) show low magnification of H & E-stained transplants after 2 weeks; middle 3 panels (D, E, F) show anti-CD3 staining. Note that the most intense and wide-spread CD3+ staining is found around and in the SSM2 tumor cells in the 129SvEv WT host. The lower 3 panels (G, H, I) are high magnification of H & E-stained areas of A, B and C, respectively. In G, arrowheads indicate monocytes suggesting a general mononuclear response in the 129SvEv WT. In H, arrows point to granulocytes and suggest that the SSM2 tumors have a general granulocytic host response in the *Stat1-null*. In I, the presence of monocytes (arrowhead) and granulocytes (arrow) suggests an intermediate immune response in *Stat1-null* hormone treated (PDOM) hosts.

To further define differences in the host response to mammary epithelium, a 129:*Stat1-null* epithelial tumor cell line SSM2^UCD^ [39] that had been transplanted into 129SvEv WT and 129:*Stat1-null* gland-intact MFPs was examined by histology and immunohistochemistry (Fig 7). SSM2 ^UCD^ tumors transplanted into the 129:*Stat1-null* MG showed a consistent and characteristic granulocytic infiltrate with rare CD3+ cells and other scattered mononuclear cells (Fig 7H). In contrast, transplants into 129SvEv WT MG were characterized by mononuclear, predominantly CD3+ lymphocytic infiltrates with relatively sparse granulocytes (Fig 7G). However, 129:*Stat1-null* hosts that were pretreated for two weeks with PDOM and then immediately implanted with SSM2^UCD^ cells showed an intermediate response with a mononuclear host infiltrate with more CD3+ cells and decreased granulocytes (Fig 7I). These host responses to transplanted tumors are consistent with the known immunological imbalance in 129:*Stat1-null* mice [22] and the expression microarray analysis of the respective intact mammary fat pads which show increased levels of granulocyte related RNA (Fig 4).

### 129:*Stat1-null* Mammary Fat Pads are Growth Factor Deficient

Transplantation of both neoplastic and normal mammary epithelium into 129:*Stat1-null* hosts resulted in retarded cell growth relative to that in the 129SvEv WT host, suggesting that the 129:*Stat1-null* microenvironment might be deficient in one or more growth-regulating factors. We first determined the growth response of SSM2^UCD^ cells and COMMA-1D cells (a phenotypically normal BALB/c mammary epithelial cell line [40]) to CM prepared using ECFPs from 129SvEv WT control, 129:*Stat1-null*, and “rescued” 129:*Stat1-null* mice treated with PDOM.

**Fig 8:**
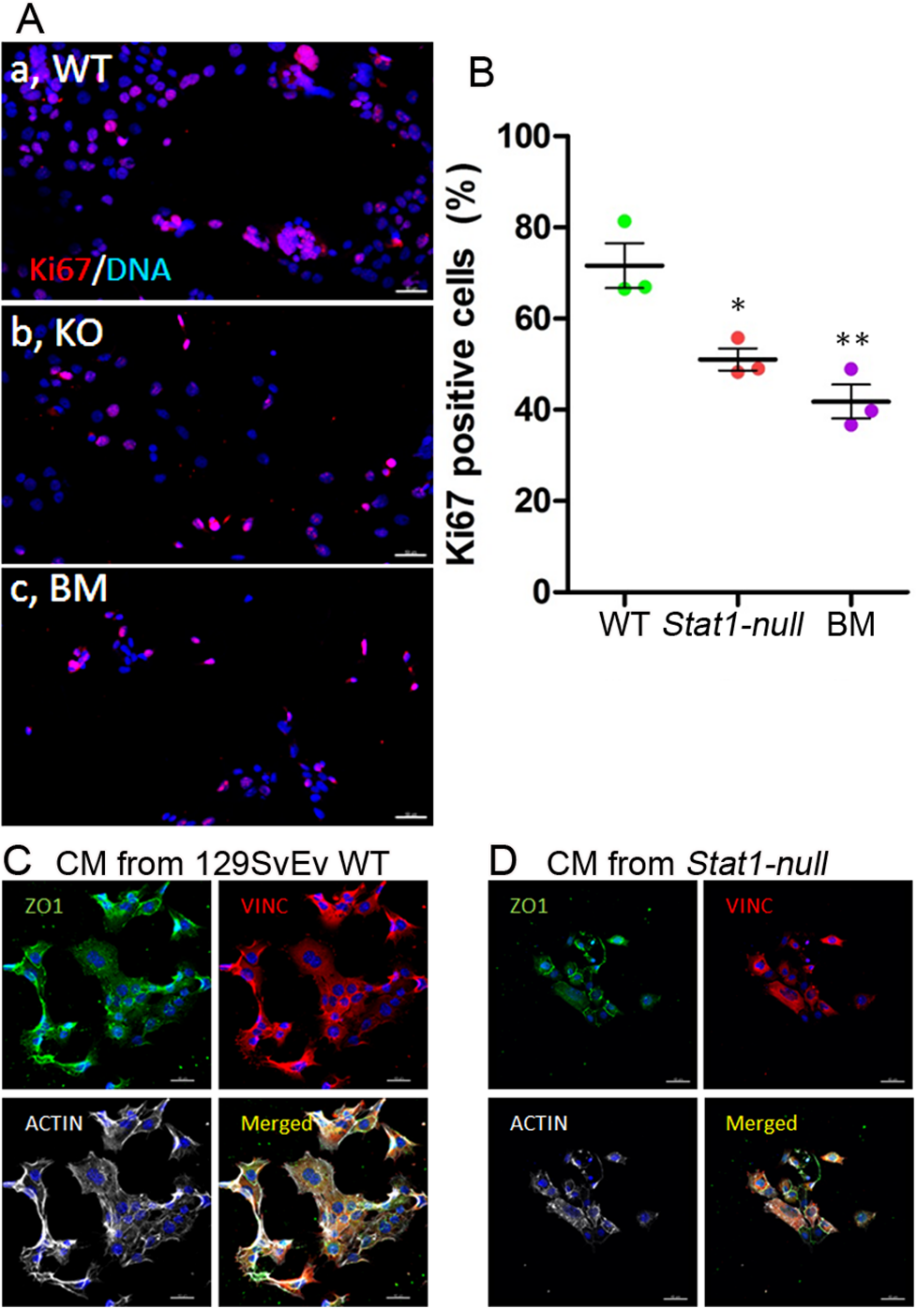
*Stat1-null* fat pad conditioned media poorly supports tumor cell line proliferation. Cell proliferation of SSM2^UCD^ cells treated with CM from epithelium-cleared fat pads (ECFP) of 129SvEv WT and 129:*Stat1-null* (*Stat1-null*) mice is demonstrated with Ki67 staining (A). Immunofluorescence staining for Ki67 (red) and DAPI (blue) shows the growth proportion of SSM2^UCD^ cells cultured in conditioned media (CM) from 129SvEv WT ECFP (a, WT), CM from *Stat1-null* ECFP (b, KO) or basal media (c, BM). The percentage of Ki67 positive cells was higher in 129SvEv WT CM than *Stat1-null* CM or BM (B). Data are mean ± SEM (n = 3). *P<0.05, **P<0.01. Immunofluorescence for ZO1 (green), VINC (red), ACTIN (white) and DAPI (blue) in SSM2^UCD^ cells cultured in CM from 129SvEv WT (C) or CM from *Stat1-null* ECFP (D) shows changes in cytological distribution of structural proteins. Scale bar = 50 μm.

The SSM2^UCD^ cells grown in 129SvEv WT CM were assessed by immunofluorescence for Ki67 (Fig 8A) that revealed a higher growth rate compared to those grown in 129:*Stat1-null* CM or basal media (Fig 8B). The SSM2^UCD^ cells cultured in 129:*Stat1-null* CM showed less cohesion compared to cells cultured with CM prepared from the MG of 129SvEv WT mice (Fig 8C and 8D, merged). SSM2^UCD^ cell clusters were more extensive when cultured in 129SvEv WT compared to 129:*Stat1-null* CM with distinctions in cell-cell junctions (ZO1 staining), focal adhesions (vinculin staining), and organization of the actin cytoskeleton (Fig 8C and 8D).

**Fig 9:**
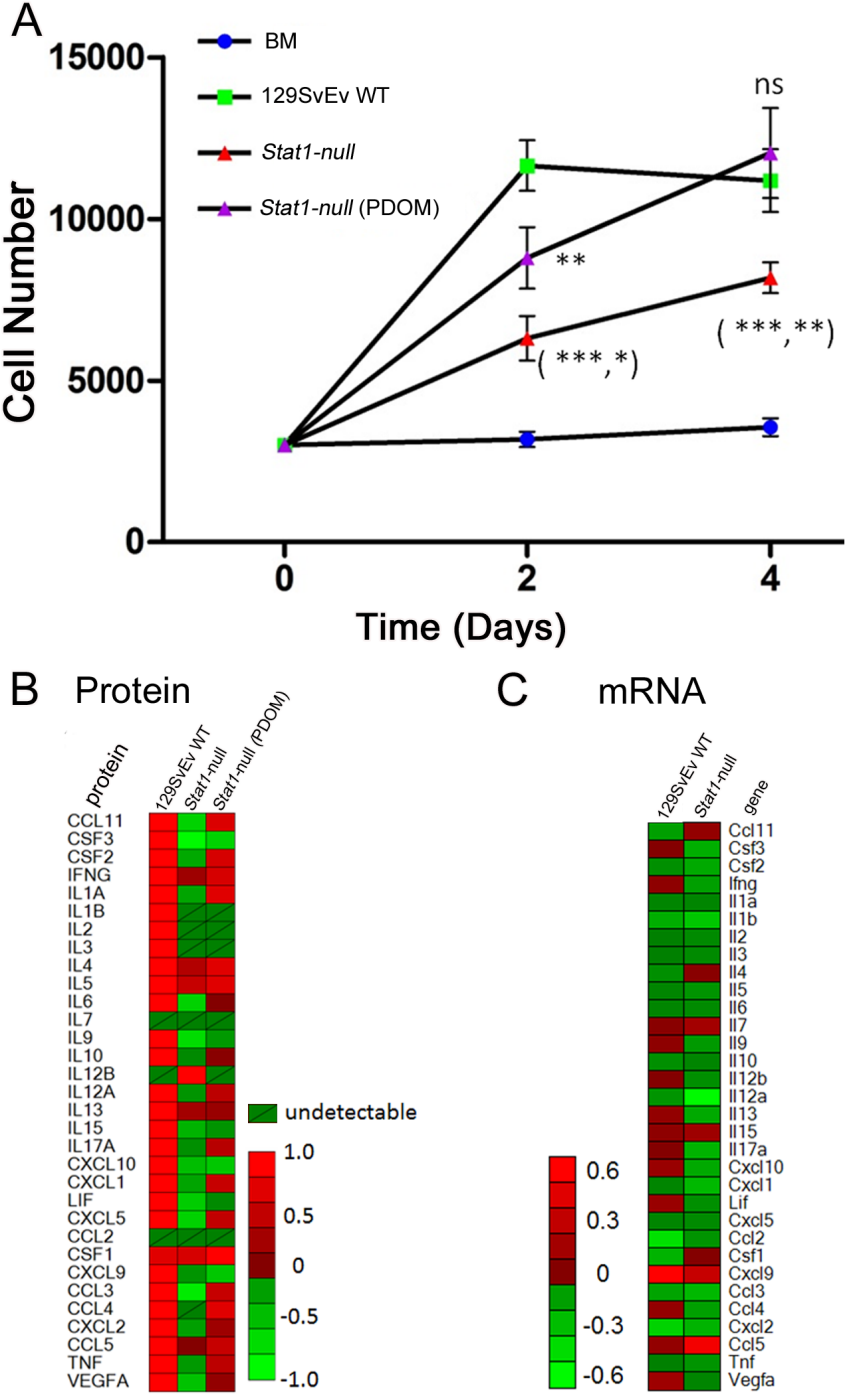
129:*Stat1-null* fat pad conditioned media inhibits growth of normal epithelium. OMMA-1D cell proliferation over time (A). Cells were grown in media conditioned by epithelium-free fat pads (ECFP) taken from either 129SvEv WT, 129:*Stat1-null* (*Stat1-null*) or PDOM-treated *Stat1-null* mice, or else basal media (BM) for 4 d. Cell proliferation was measured with a methylene blue stain assay at day 2 and day 4. Data are mean ± SEM (n = 6 wells per treatment). For 129SvEv WT vs *Stat1-null*, ***P<0.001 (day 2), **P<0.01 (day 4). For 129SvEv WT vs *Stat1-null* (PDOM), **P<0.01 (day 2). For *Stat1-null* vs *Stat1-null* (PDOM), *P<0.05 (day 2) and **P<0.01 (day 4). Data are representative of 4 separate experiments. Medium conditioned by ECFP from 129SvEv WT or *Stat1-null* mice were used to assess cytokines by Milliplex assay (B). The heat map shows the cytokine levels of CM made with ECFP from 129SvEv WT, *Stat1-null*, or *Stat1-null* mice treated with PDOM. *Stat1-null* gland-intact mammary fat pads (MFP) have lower levels of cytokine expression than 129SvEv WT gland-intact MFP (C). Heat maps of multiplex cytokine/chemokine assay results show the differences in cytokine expression levels between 129SvEv WT and *Stat1-null* gland-intact MFP.

COMMA-1D cells showed minimal growth in 129:*Stat1-null* CM when compared to their growth in serum-free basal media (Fig 9A). In contrast, the CM from 129SvEv WT ECFPs stimulated growth of COMMA-1D cells [41], with a 24 h doubling time of 2 d (Fig 9A). Interestingly, the CM from 129:*Stat1-null* ECFPs pretreated with PDOM stimulated the growth of COMMA-1D cells to an extent similar to their response to 129SvEv WT CM (Fig 9A).

### Profiles of Conditioned Media Indicate Reduced Cytokine Levels in the *Stat1-null* Mammary Fat Pad

Cell-free CM stimulated differential growth and morphological phenotypes of epithelial cells, suggesting a role for diffusible molecules such as chemokines/cytokines from the MFP. The gene expression profiles from intact MFP, alongside the results from the multiplexed protein analysis of ECFP CM, showed reduced levels of many cytokines in the 129:*Stat1-null* MFP compared with the 129SvEv WT MFP, including G-CSF, IL-6, MSP1, KC, and MIP2 (Fig 9B). Other cytokine levels in the 129:*Stat1-null* CM, such as CCL11 (eotaxin), were similar to those in 129SvEv WT CM. Of note, the medium conditioned by ECFPs from PDOM-treated mice exhibited cytokine profiles much closer to that from 129SvEv WT than 129:*Stat1-null* CM. Notably, there was a greater than 50-fold difference in CCL4 (MIP-1β) between *Stat1-null* CM and PDOM pretreated 129:*Stat1-null* CM, or 129SvEv WT CM (Fig 9B). These results indicate that the microenvironment of the ECFP in 129:*Stat1-null* mice is deficient for a large range of growth-regulatory molecules that are restored in response to PDOM.

## Discussion

Our studies document a number of developmental abnormalities in MG of nulliparous 129:*Stat1-null* mice, including delayed ductal elongation and defective branching morphogenesis that could be rescued with pregnancy and lactation. These structural abnormalities are accompanied by abnormal, enlarged terminal end buds with disorganized clusters of cells in the surrounding stroma. Subsequently, 129:*Stat1-null* mice were found to have low levels of cytokines that likely explains the inability of the MFP to sustain growth of primary and transplanted mammary epithelial normal and neoplastic cells. The growth-limiting deficit in the 129:*Stat1-null* MFP can, in part, be overcome with pregnancy or exogenous PDOM that restores the cytokine profile within the MFP and reverses the epithelial growth and development defects. However, the loss of STAT1 is also associated with minor, but statistically significant, PDOM-resistant abnormality in branching morphogenesis that suggests an epithelial cell-autonomous defect These phenomena highlight multiple potential roles for STAT1 in regulating the complex interplay between the endocrine and stromal environments during MG development.

The comparison of MG development in 129SvEv WT and 129:*Stat1-null* females also reveals clear morphological differences between the genotypes. The 3D structural analysis of whole mounts from six-week-old mice documents that the 129:*Stat1-null* mice have larger TEBs and a widely-dispersed overabundance of adjacent stromal “escort cells”. The presence of these large TEB surrounded by scattered small stromal cells suggests that the signals necessary to organize and coordinate the complex interactions between stroma and epithelium during ductal extension may be weak or missing in *null* mice.

The MGs of mature virgin 129:*Stat1-null* females are also underdeveloped with reduced ductal branching that persisted beyond sexual maturity. However, the 129:*Stat1-null* females can successfully nurse pups with structurally normal and functional MGs during lactation, indicating that the phenotypic abnormality is reversible with hormone stimulation.

Given the global role of JAK-STAT [33] in the MGs of nulliparous females and during post-weaning involution [42], it is somewhat surprising that previous studies did not perform detailed analyses of MG development, but chose to primarily focus on tumorigenesis [12–15]. This discrepancy was also intimated by Haricharan and Li [6]. Neither Raven et al. nor Chan et al. specifically presented data on the *Stat1-null* non-neoplastic MGs [12, 14]. When evaluating MG whole mounts from 50-day-old nulliparous females, Schneckenleithner et al. described”…an increased density of ductal structures but no differences in end duct formation” and showed the normal histology in a regressing Balb/c wild type MG [15]. In contrast, Klover et al. did not report significant differences in the MGs of their epithelial-specific FVB:*Stat1-null* mice, thereby implying there were no cell-intrinsic abnormalities [13]. However, the image presented by Klover et al. as evidence for their statement that “A complete lack of STAT1 expression is observed in Stat1fl/fl NIC mice” was illustrated in Fig 4 in “Non–tumor-containing epithelium from approximately 1 year-old tumor-bearing mice is shown.” The epithelium illustrated was identified as hyperplastic and perhaps neoplastic by our experienced pathologists. Further evidence was based on a WAP-Cre knockout that in and of itself would require a pregnancy for promoter activation, which, as demonstrated in the current paper, obliterates the *Stat1-null* MG phenotype.

Differences between our present findings and the latter two reports could be due to differences in constructs, strains and genetic background of mouse models or other factors [43]. The critical role of STAT1 in signal transduction in multiple organ systems [33] prompted us to undertake a more detailed study of MG development in 129:*Stat1-null* females.

STAT1 is responsible for IFNγ signaling [6] and also mediates PRL signaling [44–46]. STATs 1, 3 and 5 are activated by a variety of extracellular stimuli including growth factors, hormones and cytokines [6, 42]. Empirically, pregnancy rescued the 129:*Stat1-null* MG morphological phenotype, and exogenous PDOM increased growth of null epithelium in the 129:*Stat1-null* hosts to an extent approaching that recorded in 129SvEv WT hosts. Significantly, full recovery of MG growth and development required a combination of ovarian (P) and pituitary (PRL) hormones, which could not be realized with the individual hormones (S1 Fig). However, the 129:*Stat1-null* epithelium still demonstrated statistically significantly reduced ductal branching, even after hormone stimulation and growth in a 129SvEv WT host (Fig 3). This finding suggests that, while hormones can stimulate the 129:*Stat1-null* epithelium, the subsequent branching abnormalities are, in part, epithelial-cell autonomous.

Reciprocal MG transplants revealed that both 129SvEv WT and 129:*Stat1-null* epithelium could grow in 129SvEv WT hosts, whereas the syngeneic 129:*Stat1-null* hosts did not support the same level of growth and differentiation of either epithelial genotype. STAT1 deficient mice are immunodeficient and are susceptible to *Listeria* and *plasmodium* infections [17, 21, 22] and have been used to study immune editing during tumorigenesis [22, 35], raising the possibility that reduced MG growth in 129:*Stat1-null* females reflected systemic changes in marrow-related cells.

Bone marrow-derived immune cells, particularly monocyte/macrophages, are important for mammary duct elongation at the terminal end bud [37, 47]. However, reciprocal bone marrow transplantation demonstrated that the impaired MG development in nulliparous 129:*Stat1-null* mice is not dependent upon the immune cells themselves, but is more likely to reflect a defect in recruitment of these cells by an altered microenvironment. The epithelial response to these stromal abnormalities is also not limited to “normal” 129:*Stat1-null* epithelial cells given that 129:*Stat1-null* neoplastic cells transplanted into both 129SvEv WT and 129:*Stat1*-*null* hosts grew more rapidly in 129SvEv WT hosts. Notably, the early 129:*Stat1*-*null* host response to the tumor cells was characteristically granulocytic while the early host response in 129SvEv WT was mononuclear and dominated by CD3+ T-cells. Such observations are consistent with known immunological deficiencies in the 129:*Stat1*-*null* mice having defects in the function of NK cells and macrophages [35, 36], and further confirm that the MG microenvironment is altered between WT and 129:*Stat1-null* mice.

The biological complexity of the JAK-STAT interactions should also influence the interpretation of tumorigenesis in nulliparous and parous 129:*Stat1-null* females in comparison to other STAT1 models. The 129:*Stat1-null* females develop mammary tumors having a unique ER+, progesterone receptor (PR)+ histological signature distinct from other models [12, 38]. Prior studies of mammary tumorigenesis in *Stat1-null* mice in other mouse strains [13–15] and using different molecular constructs [48] have induced tumors either by crossing mice with tumor-prone cNeu transgenic mice [13–15] or by pregnancy [15]. In contrast, tumors develop spontaneously in 129:*Stat1-null* nulliparous females [12].

Using transplants of normal mammary epithelium coupled with parity induction of tumorigenesis in the Balb/c model, Schneckenleithner et al. suggested that the immune response and cell-intrinsic factors are important during tumorigenesis induced by loss of STAT1 [15]. Our data are consistent with these general conclusions. However, the histopathology of the spontaneous ovarian dependent tumors in the 129:*Stat1-null* MG is homogeneous with a unique cytological ER+ phenotype [12]. In contrast, the “spontaneous” precancerous lesions (referred to as “MIN”) and tumors in the parity-induced Balb/c model are described as heterogeneous [15]. Unfortunately, the descriptions of those lesions used human-based classifications that are difficult to translate to traditional or current mouse mammary tumor classifications [49–51]. Examination of the images in the Schneckenleithner et al. publication suggests that the heterogeneous tumors bear hallmarks consistent with those previously documented in animals infected with Mouse Mammary Tumor Virus (MMTV) [49, 52, 53] and described in genetically engineered mice with perturbation of the Wnt pathway [54]. Since the WT multiparous Balb/c mice used in those studies also developed mammary tumors, it is possible that the colony is expressing the nodule inducing virus (NIV) type of MMTV found in C3Hf and some BALB/c colonies [53], which would further confound comparisons between the different *Stat1*-null models.

Profiling of the cytokines within three types of cell-free medium conditioned by cultured explants of MFP revealed a global and profound diminution of cytokines in the 129:*Stat1*-*null* CM. Notably, the cytokine expression profiles from intact 129:*Stat1*-*null* MGs were comparable to those of the CM prepared using only the ECFP. Of note, the cytokines eotaxin (CCL11), CSF, and MIP-1α (CCL3) are related to eosinophil or macrophage functions, which aligns with the short-term (14 d) immunological responses within transplants of 129:*Stat1*-*null* tumors into 129SvEv WT and 129:*Stat1-null* hosts.

We, and others, previously showed that diffusible unsaturated fatty acids are a major component of the mitogenic capacity of CM prepared using explants of MFP [41, 55]. This consideration raises the question of whether the metabolic capacity of the 129:*Stat1-null* MFP is modified, and whether this property is affected by the altered profile of microenvironmental cytokines. Certainly STAT1 is crucial for adipocyte function where it mediates IFNγ-regulated lipolysis in adipocytes [56] and the effects of prostaglandins on their differentiation [57]. At the same time we found that both the cytokine profile and mitogenic capacity of CM could be restored by exogenous PDOM. We [32, 58] and others [59] have highlighted the potential for convergence of P and PRL signaling in the MG, where P alone can stimulate ductal development in the MG [60, 61], likely via synergy with IGF-1 [62]. Along these same lines, we showed that P and PRL synergized to stimulate proliferation of the mammary ductal epithelium of mice independent of E [32]. Furthermore, P and PRL are both known to signal via STAT1 [46, 63], and both P and PRL can affect the activity of adipose tissue and its local production of cytokines [64–66]. Thus, an evolving hypothesis is that the nulliparous 129:*Stat1-null* MFP lacks diffusible molecules such as cytokines and/or fatty acids that are required to sustain normal mammary growth and development, and that these are locally mediated by endocrine cues during ductal elongation. Further studies are required to dissect this complex hormonal, tissue, cellular and molecular milieu and are beyond the scope of this paper.

Finally, all these factors will need to be considered in any study of tumorigenesis in STAT1 deficient mice, where our data indicate that aberrant MG development directed by the 129:*Stat1-null* microenvironment leads to increased tumor initiation. Since 129:*Stat1-null* mice model neoplastic development of ER-positive mammary tumors later in human life, the model warrants further attention. A major challenge will be to determine how a neoplastic cell arises and how it continues to grow in the relatively unsupportive 129:*Stat1*-*null* growth microenvironment. At this stage it seems unlikely that simple cell-autonomous over-expression of growth-promoting genes can sufficiently explain neoplastic progression. The enigma that remains to be solved is which tumor cell-host microenvironment interactions are at play during tumor initiation versus progression.

## Supporting Information

S1 Fig Whole mount images of 129SvEv WT and 129:*Stat1-null* mammary glands during lactation, pre-lactation and involution. (TIF)

S2 Fig Low power whole mount images of mammary glands from Fig 2A. (TIF)

S3 Fig Circulating hormone levels of 129SvEv WT and 129:*Stat1-null* mice. (TIF)

S4 Fig Estrogen (E) or progesterone plus domperidone (PDOM), restores branching in 129:*Stat1-null* mammary gland. (TIF)

S5 Fig 129:*Stat1-null* mammary gland expression microarray profiles indicate altered regulation of Stats and Jaks. (TIF)

S1 Table Mammary gland epithelium transplant take rate. (TIF)

S2 Table Student's t test P value (one-tail) comparisons among transplant groups. (TIF)

S1 Text Materials and methods for qPCR for bone marrow experiments. (DOC)
