# Supplementary figures and images for: Abnormal Mammary Development in 129:STAT1-Null Mice is Stroma-Dependent
Source: PLoS One. 2015 Jun 15;10(6):e0129895. doi: 10.1371/journal.pone.0129895 (PMC4468083; doi:10.1371/journal.pone.0129895)

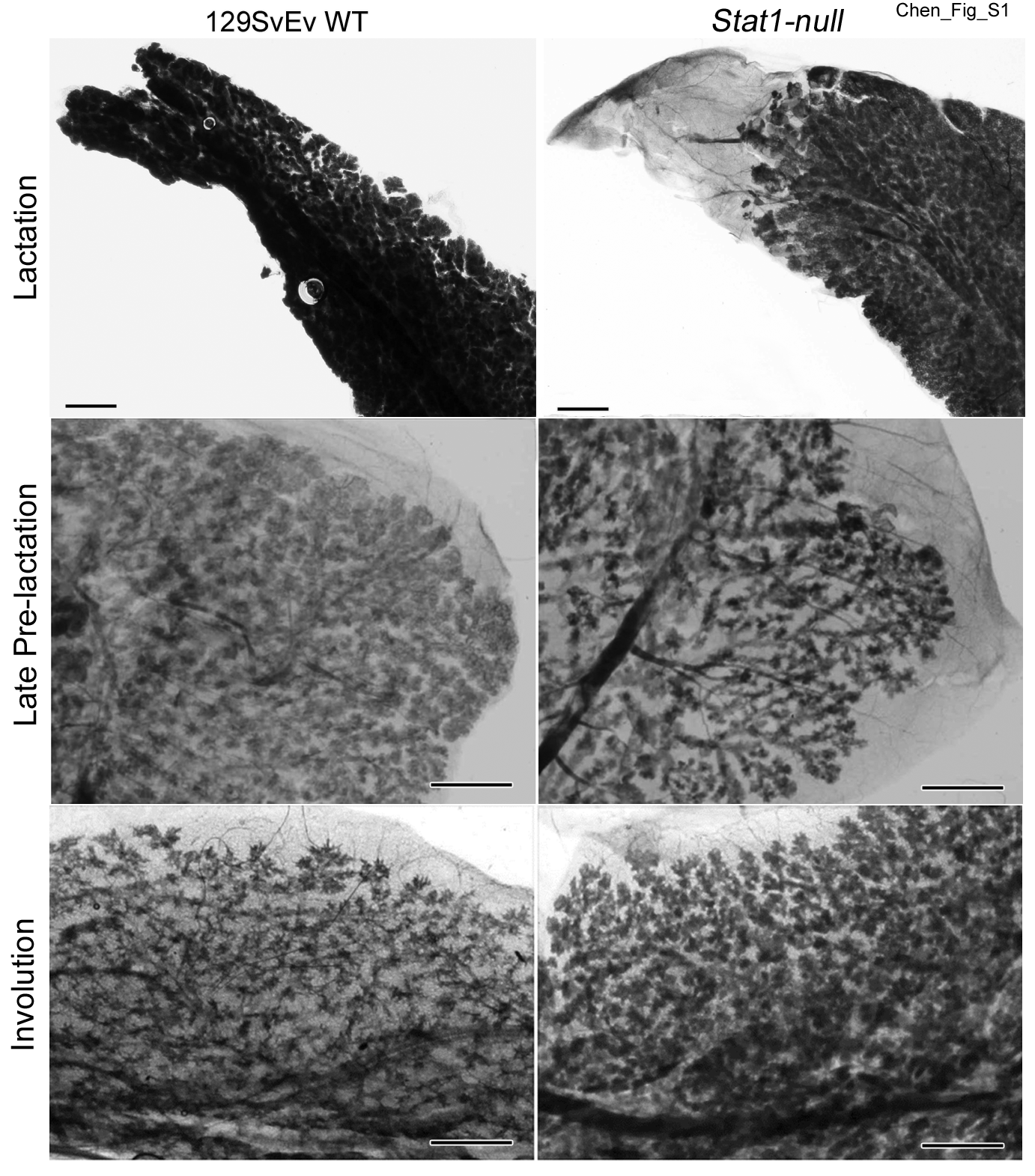

Supplement: S1 Fig — (TIF) [file pone.0129895.s001.tif]

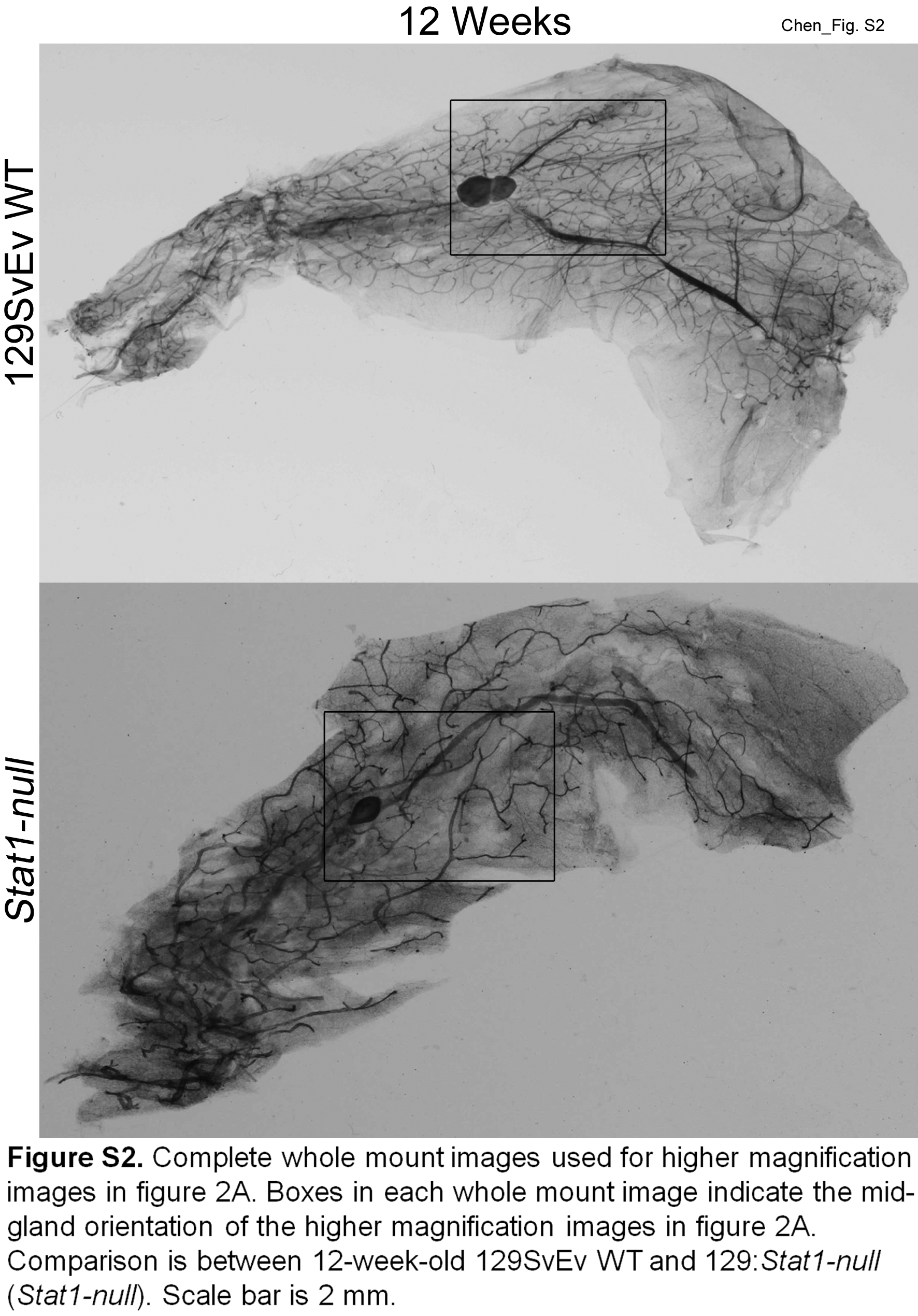

Supplement: S2 Fig — (TIF) [file pone.0129895.s002.tif]

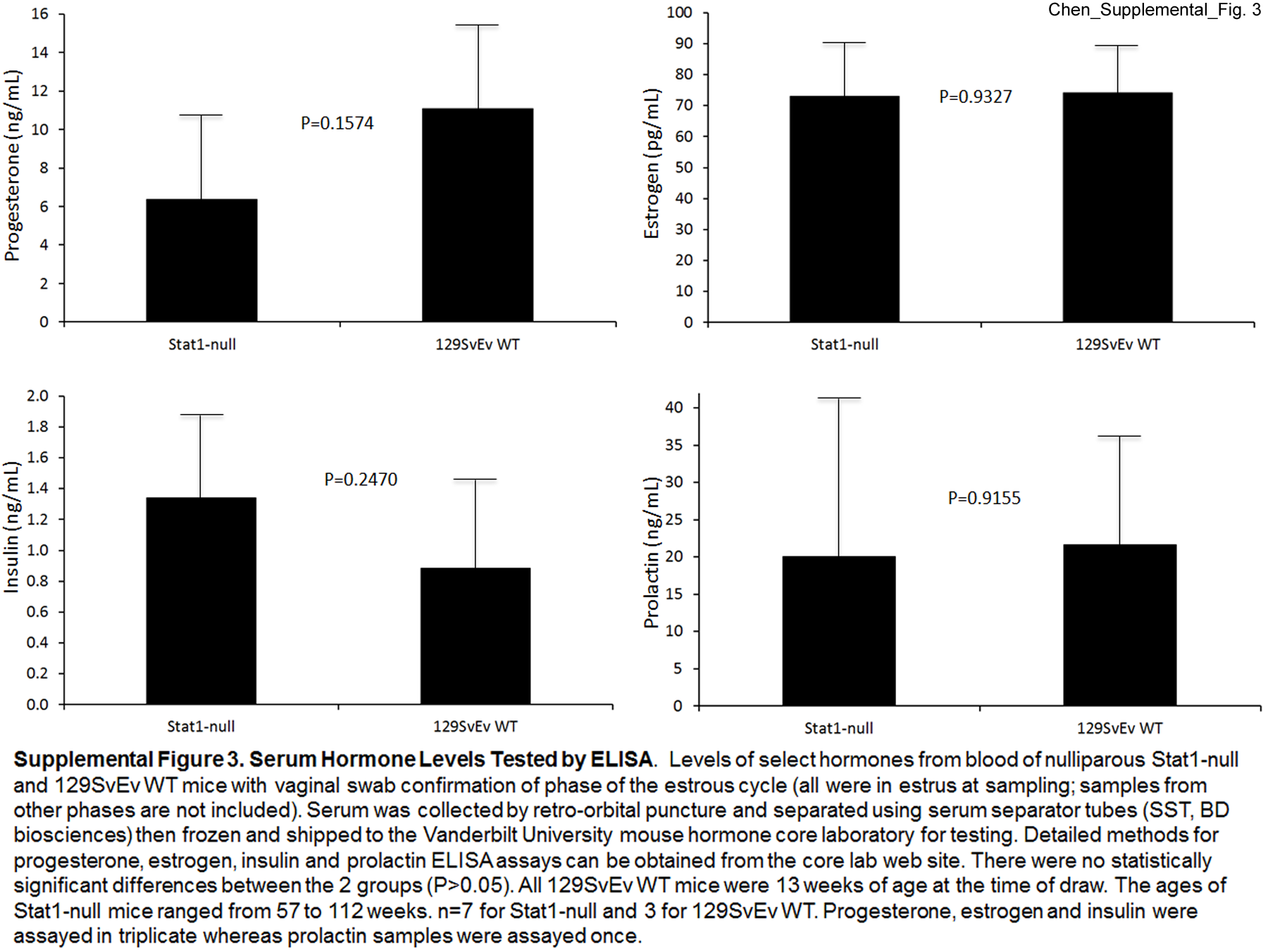

Supplement: S3 Fig — (TIF) [file pone.0129895.s003.tif]

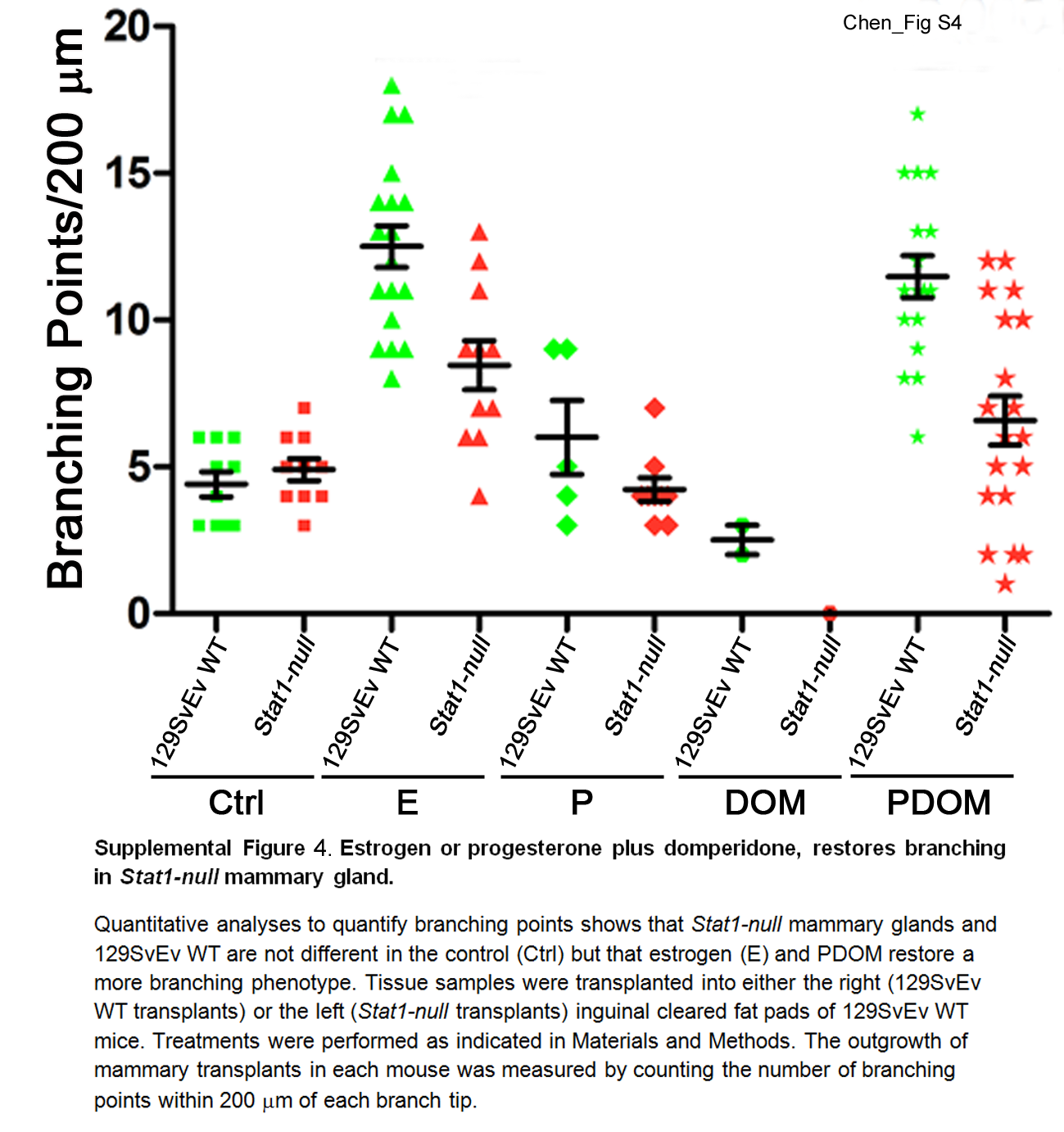

Supplement: S4 Fig — (TIF) [file pone.0129895.s004.tif]

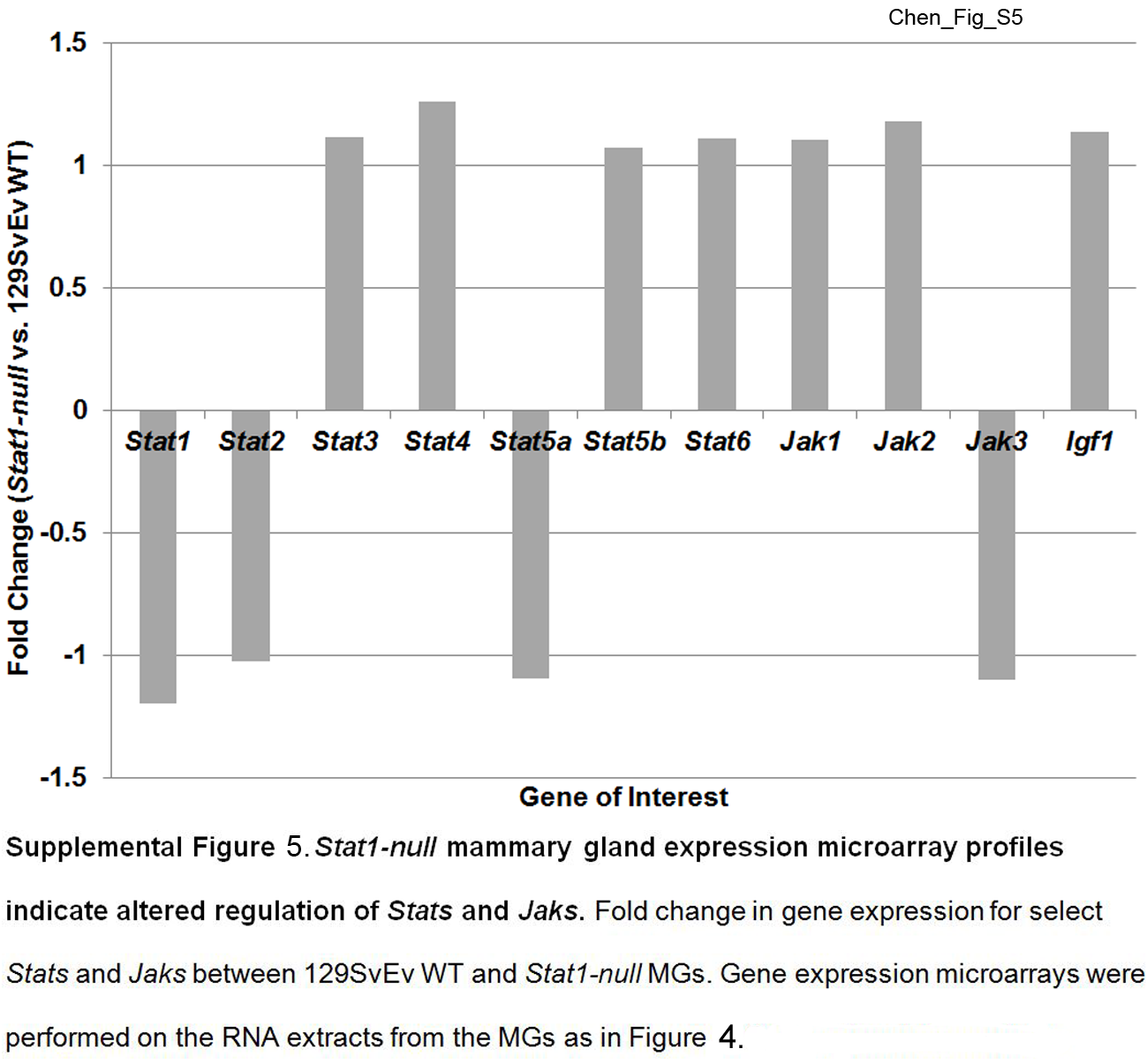

Supplement: S5 Fig — (TIF) [file pone.0129895.s005.tif]

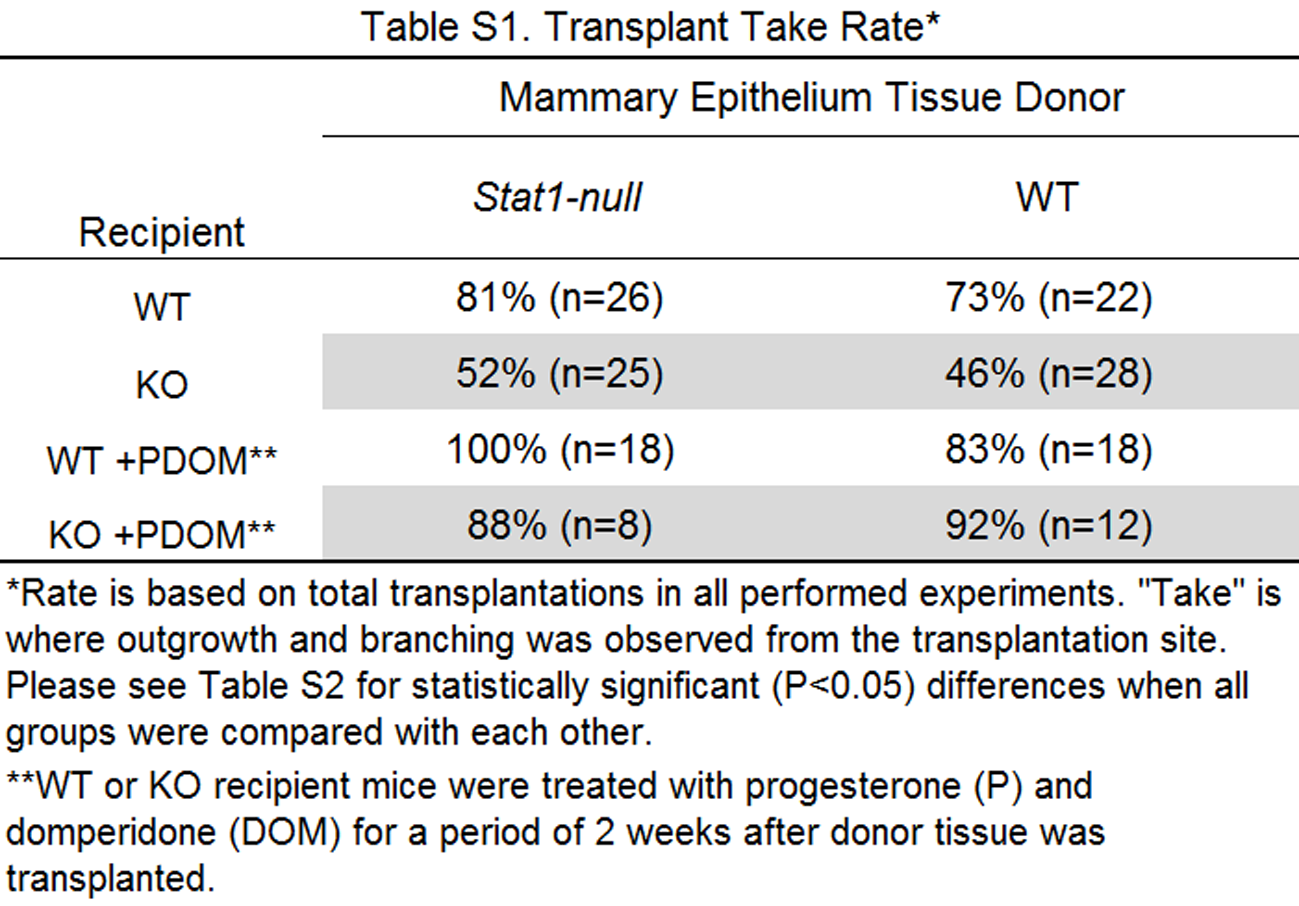

Supplement: S1 Table — (TIF) [file pone.0129895.s006.tif]

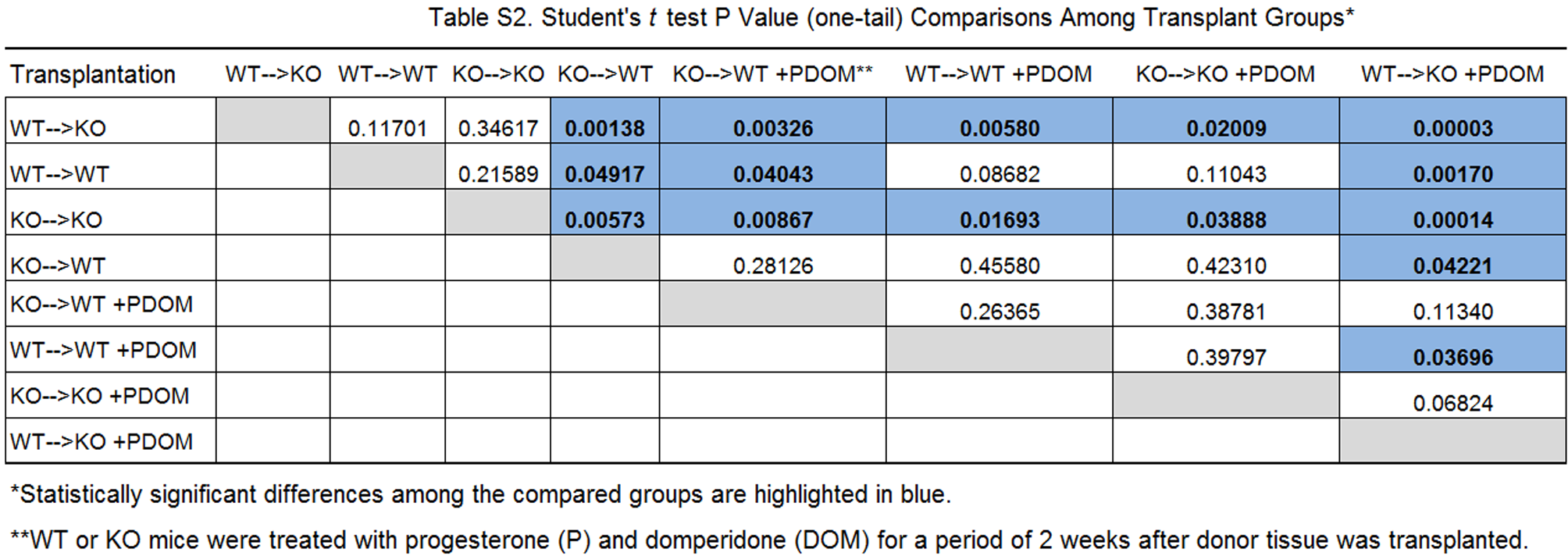

Supplement: S2 Table — (TIF) [file pone.0129895.s007.tif]
