# Supplementary material for: Abnormal Mammary Development in 129:STAT1-Null Mice is Stroma-Dependent
Source: PLoS One. 2015 Jun 15;10(6):e0129895. doi: 10.1371/journal.pone.0129895 (PMC4468083; doi:10.1371/journal.pone.0129895)
Supplement: S1 Text — (DOC) [file pone.0129895.s008.doc]

Supplemental Materials and Methods

qPCR of gDNA. Genomic DNA was extracted from pelleted erythrocytes using Qiagen DNeasy blood & tissue kit following manufacturer’s protocol. DNA was then assayed using Taqman® quantitative PCR via the multiplexed relative cycle threshold (Ct) method. Briefly, 2 μL of each DNA sample was tested in a total reaction volume of 10 μL in quadruplicate utilizing Qiagen QuantiTect PCR kit with ROX chemistry. 384-well plates were processed on an AB7900HT with the following cycles: 95°C for 15 min and then 40 cycles of 95°C for 30 sec, 60°C for 1 min. Two Taqman® assays were designed and utilized including a Neomycin selection cassette region from the targeting vector in addition to a native *Stat1-null* region removed from the targeted Stat1 locus. Mutant sequence (Neo) primers/probe used included a forward primer CCATTCGACCACCAAGCG, probe 6FAM-AACATCGCATCGAGC-MGB/NFQ, and reverse primer AAGACCGGCTTCCATCCG. Native *Stat1-null* region primers/probe used included a forward primer CGCTGGCTAGGGAGTAGAGT, probe 6FAM-TTGTTGATGAAGTAGCTTGGAGATGTGTT-NFQ, and reverse primer ATGGCCACTGGTGATAGTCAC. Each of the Neo and Stat1 sequences were multiplexed and quantified relative to an endogenous reference (ultra-conserved 329) with primers/probe including a forward primer GTCATCAAGTGAGAAAGACATCCT, probe VIC-CTCCTGGCTGCCTG-MGB/NFQ, and reverse primer CATCATGAATTTTGATAAGCCCATT. Each DNA sample averaged ΔCt was compared against the averaged ΔCt of either a homozygous *Stat1-null* DNA (Neo assay) or 129SvEv WT DNA (native Stat1 KO assay) producing a ΔΔCt. Percentage of *Stat1-null* derived erythrocyte cells was calculated from the native Stat1 KO assay as % = 1-2-(ΔCtErythrocyte-ΔCtWildtype) where % is expressed as a decimal. Inversely, the Neo assay ΔΔCt comparison between erythrocyte DNA and a homozygous *Stat1-null* DNA was calculated as % = 2-(ΔCtErythrocyte-ΔCtHom) where % is expressed as a decimal. Both assays showed a congruent and strong correlation that due to the majority of erythrocyte cells being derived from the *Stat1-null* cells, we used the more accurate Native Stat1 KO assay calculations. *Stat1-null* derived erythrocyte cells accounted for an average of 77.8% of the erythrocyte cell DNA assayed (n=7, Stdev=0.098).
